# Manipulating energy transfer in lanthanide-doped single nanoparticles for highly enhanced upconverting luminescence†
†Electronic supplementary information (ESI) available: Supplementary experimental details, Fig. S1–22 and Table S1. See DOI: 10.1039/c7sc01393k


**DOI:** 10.1039/c7sc01393k

**Published:** 2017-04-21

**Authors:** Zhu Zhuo, Yongsheng Liu, Dajiu Liu, Ping Huang, Feilong Jiang, Xueyuan Chen, Maochun Hong

**Affiliations:** a CAS Key Laboratory of Design and Assembly of Functional Nanostructures , Key Laboratory of Optoelectronic Materials Chemistry and Physics , State Key Laboratory of Structural Chemistry , Fujian Institute of Research on the Structure of Matter , Chinese Academy of Sciences , Fuzhou , Fujian 350002 , China . Email: liuysh@fjirsm.ac.cn ; Email: xchen@fjirsm.ac.cn ; Email: hmc@fjirsm.ac.cn; b University of the Chinese Academy of Sciences , Beijing , 100049 , China

## Abstract

Energy transfer (ET) is of fundamental importance in tuning the optical performance of lanthanide-doped upconversion nanoparticles (UCNPs). However, the fine control and manipulation of the ETs particularly for deleterious cross-relaxation type ETs (CR-ETs) in lanthanide-doped UCNPs remains a formidable challenge to date. Herein, we demonstrate a rational design strategy to manipulate the deleterious CR-ETs in lanthanide-doped UCNPs, by fine-tuning the distances at an extremely large length scale (>20 nm) among multiple lanthanide dopants that are simultaneously embedded into one single nanoparticle with specially designed multilayer nanostructures. The successful inhibition of the CR-ETs leads to a significantly enhanced upconversion luminescence signal with an intensity ∼70 times higher than that of co-doped conventional UCNPs. This finding paves a new way for the better control of the ETs in lanthanide-doped nanoparticles, and offers the possibility of constructing a series of promising single-nanocrystal-based anti-counterfeiting barcodes with well-identified UC emission color and lifetime outputs.

## Abstract 2

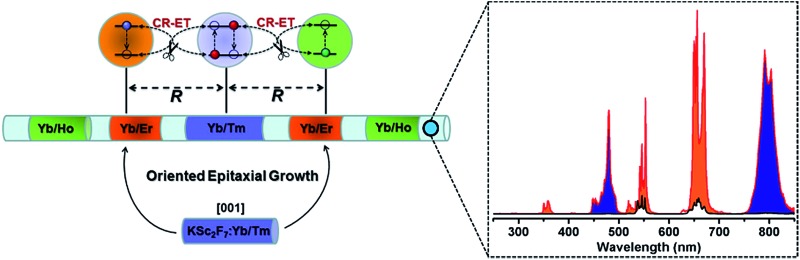
We demonstrate a multilayer-structured design strategy to manipulate the deleterious CR-ETs in lanthanide-doped UCNPs for highly enhanced upconverting luminescence.

## Introduction

Trivalent lanthanide (Ln^3+^)-doped upconversion (UC) nanoparticles (UCNPs) that convert low-energy irradiation into high-energy emission are promising for applications in diverse fields such as biological detection, imaging, therapeutics, multiplexed encoding and three-dimensional full-color displays.^1^–^19^ Although recent advances in the synthesis of Ln^3+^-doped UCNPs have allowed for the fine-tuning of nanoparticle size, composition, morphology, phase, structure as well as UC emission colors, there is still much room for improvement in their UC luminescence efficiency particularly for those UCNPs co-doped with multiple Ln^3+^ ions.^20^–^30^ For instance, limited by their intrinsic electronic configurations, typical UC activators including Er^3+^, Tm^3+^ and Ho^3+^ ions are usually co-doped into the lattices of diverse UCNPs coupled with the sensitizer of Yb^3+^ ions to yield multicolor emissions spanning from the ultraviolet (UV) to near-infrared (NIR) spectral regions.^31^,^32^ However, such a co-doping strategy can inevitably lead to an increase in the overall Ln^3+^ doping concentration in one single nanoparticle, thereby giving rise to serious concentration quenching of the UC luminescence (ESI Fig. S1†).^33^ In principle, such UC luminescence quenching occurring at high Ln^3+^ ion doping levels is primarily associated with the unwanted cross-relaxation type energy transfers (CR-ETs) among the neighboring Ln^3+^ ions, which may quench the excitation energy and dissipate it non-radiatively as heat in the lattices of the UCNPs and thus result in weak UC emissions.^34^,^35^ In this regard, the precise control of such unwanted CR-ETs in the UCNPs holds great promise in getting to the root of the concentration quenching, which hitherto remains a formidable challenge. It is well known that the probability of CR-ETs for two given neighboring Ln^3+^ ions is strongly dependent on their spatial distance.^33^ Therefore, the most straightforward and feasible way to manipulate the CR-ETs is to control the distances among the Ln^3+^ ions that are embedded in one identical nanoparticle. Attempts to solve this problem include lowering the doping concentration of the Ln^3+^ ions or confining the doped Ln^3+^ ions in different layers of core–shell structured UCNPs.^36^–^38^ However, only a very limited distance between the Ln^3+^ emitters (typically <5 nm) can be tuned for these two approaches due to their intrinsic limitations such as elemental migration across the core–shell interfaces and the inhomogeneous growth of different shell layers for the core–shell structured UCNPs,^36^,^39^,^40^ which thereby cannot completely minimize the negative effect of the deleterious CR-ETs.

**Scheme 1:**
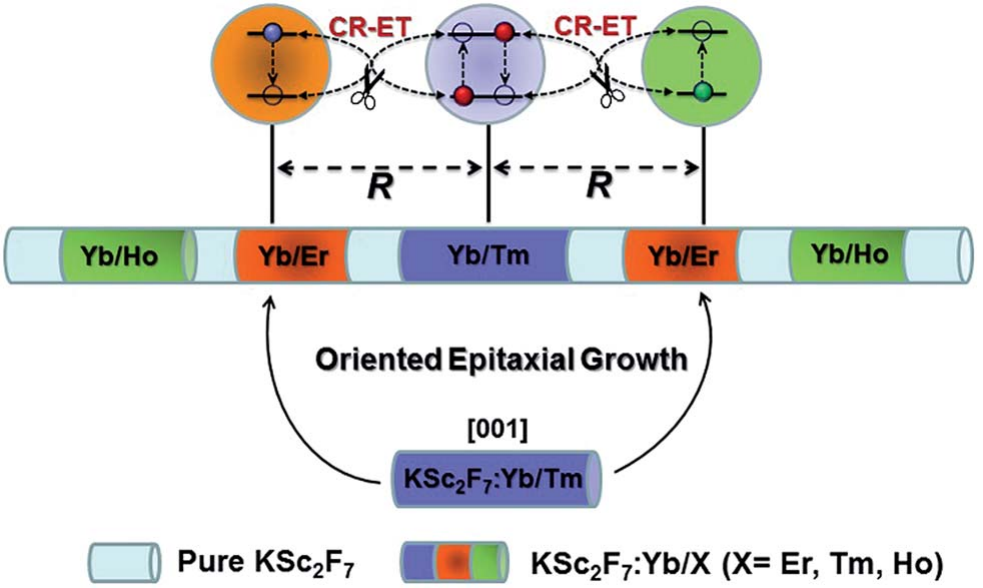
Schematic design of a multilayer-structured KSc_2_F_7_ nanorod comprising different Yb/X pairs separately incorporated in layers and thickness-tunable pure KSc_2_F_7_ interlayers to manipulate CR-ETs in one single nanorod for highly enhanced UC luminescence.

To this end, herein we demonstrate a rational design strategy to fundamentally circumvent the concentration quenching effect that is ubiquitous in Ln^3+^ ion doped UCNPs. Our strategy is based on manipulating the CR-ETs at an extremely large length scale (>20 nm) through fine-tuning the distances among the Ln^3+^ dopants that are spatially confined into different layers by inserting thickness-tunable pure host interlayers into one single nanorod with specially designed multilayer structures (Scheme 1). Benefiting from this strategy, unwanted CR-ETs can be effectively eliminated in UCNPs co-doped with multiple Ln^3+^ ions, resulting in highly enhanced UC emissions when compared to the conventional KSc_2_F_7_ UCNPs in which all Ln^3+^ ions are co-doped together. The significantly enhanced UC luminescence coupled with the flexibility of multilayer structures thereby enable us to fabricate a series of color-tunable UC nanorods that can serve as promising single-nanocrystal-based anti-counterfeiting barcodes with well-identified UC color and lifetime outputs.

## Experimental section

### Chemicals and materials

CF_3_COOH, Ln_2_O_3_ (Ln = Sc, Y, Gd, Yb, Er, Ho and Tm), Ln(CH_3_COO)_3_·4H_2_O, CF_3_COOK·H_2_O, oleic acid (OA), oleylamine (OM), and 1-octadecence (ODE) were purchased from Sigma-Aldrich (China). Ln(CF_3_COO)_3_·4H_2_O was prepared as reported in the literature.^41^ Polystyrene beads (9.0–9.9 μm) were purchased from Aladdin Reagent Co., Ltd. 1-Butanol, dichloromethane, cyclohexane, NaOH, NH_4_F, and ethanol were purchased from Sinopharm Chemical Reagent Co., China. Unless otherwise noted, all of the chemical reagents were used as received without further purification.

### Structural and optical characterization

Powder X-ray diffraction (XRD) patterns of the samples were collected using an X-ray diffractometer (MiniFlex2, Rigaku) with Cu Kα1 radiation (*λ* = 0.154187 nm). Representative transmission electron microscopy (TEM), high angle annular dark-field scanning TEM (HAADF-STEM) and electron energy loss spectroscopy (EELS) analyses were conducted using a TECNAI G2F20 TEM. The hydrodynamic diameter distribution for the ligand-free KSc_2_F_7_ nanorods dispersed in aqueous solution (pH 6.9) was determined by means of dynamic light scattering (DLS) measurement (Nano ZS ZEN3600, Malvern). UC luminescence spectra were measured upon 980 nm NIR excitation from a continuous-wave diode laser. UC luminescence decays were measured with a customized UV to mid-infrared steady-state and phosphorescence lifetime spectrometer (FSP920-C, Edinburgh Instrument) equipped with a digital oscilloscope (TDS3052B, Tektronix) and a tunable mid-band Optical Parametric Oscillator (OPO) pulse laser as the excitation source (410–2400 nm, 10 Hz, pulse width ≤ 5 ns, Vibrant 355II, OPOTEK). All of the UC luminescence decay curves were fitted using a double exponential function unless otherwise noted. The absolute UC quantum yields (QYs) for the samples were measured with a customized UC luminescence spectroscopy system at room temperature with a 980 nm diode laser (LSR-PS-II, Lasever. Inc) excitation at power densities of 20–50 W cm^–2^, and the UC emission peaks from the Er^3+^, Ho^3+^ and Tm^3+^ ions in the spectral range of 300–850 nm were integrated for the QY determination. All of the spectral data were corrected for the spectral responses of both the spectrometer and the integrating sphere. UC luminescence microscopy images for polystyrene microbeads loaded with diverse multilayer-structured KSc_2_F_7_ nanorods were obtained on a Nikon ECLIPSE Ti-U microscope with a Nikon digital sight DS-Ri1 CCD imaging system. UC luminescence photographs for the 2D code, multicolored sketch and the Arabic numbers of “123456” were printed/handwritten onto a piece of A4 paper using our multilayer-structured nanorods as UC security ink and were taken with a Canon 70D digital camera, where a short pass filter of 750 nm was placed in the front of the camera to filter the 980 nm excitation light.

### General procedure for the preparation of KSc_2_F_7_:Ln precursors

In a typical procedure, CF_3_COOK·H_2_O and Ln(CF_3_COO)_3_·4H_2_O, at a designated K/Ln mole ratio of 1 : 2, were first mixed with OA, ODE and OM with a volume ratio of 3 : 2 : 1 in a 250 mL three-neck round-bottom flask, then heated at 150 °C for 30 min under a N_2_ flow to form a clear yellowish solution, and then cooled down to room temperature naturally for the following use. It should be noted that the Ln ion, the Ln amount as well as the total solvent volume should be adjusted according to the structure of the synthesized multilayer-structured KSc_2_F_7_ nanorods.

### General procedure for the synthesis of multilayer-structured KSc_2_F_7_ nanorods

The synthesis of the multilayer-structured KSc_2_F_7_ nanorods was conducted using a modified stepwise layer-by-layer oriented epitaxial growth protocol of thermal decomposition.^42^ Taking the Tm@Er@Ho@Pure nanorods with a pure KSc_2_F_7_ interlayer thickness of ∼10.3 nm as an example, 0.5 mmol of CF_3_COOK·H_2_O, 0.79 mmol of Sc(CF_3_COO)_3_·4H_2_O, 0.2 mmol of Yb(CF_3_COO)_3_·4H_2_O and 0.01 mmol of Tm(CF_3_COO)_3_·4H_2_O were first added to a 500 mL three-neck round-bottom flask containing 15 mL of OA, 10 mL of ODE and 5 mL of OM, and then heated at 150 °C under a N_2_ flow with constant stirring for 30 min to remove the water from the raw materials. The obtained mixture was heated to 300 °C under a N_2_ flow with constant stirring and all conditions were maintained for 30 min to obtain KSc_2_F_7_:Yb/Tm seed nanorods. After retrieving 1 mL of the reaction mixture for TEM analysis, 24 mL of pure KSc_2_F_7_ interlayer precursors with a calculated amount of ∼0.5 mmol was immediately injected into the reaction mixture and ripened at 300 °C for 30 min, followed by the similar injection and ripening cycles being performed three times. Subsequently, the KSc_2_F_7_:Yb/Er, pure KSc_2_F_7_, KSc_2_F_7_:Yb/Ho and pure KSc_2_F_7_ precursors were injected and ripened in turn by using the identical injection and ripening cycles aforementioned to prepare Tm@Er@Ho@Pure nanorods with the following nanostructures: KSc_2_F_7_:Yb/Tm@KSc_2_F_7_@KSc_2_F_7_:Yb/Er@KSc_2_F_7_@KSc_2_F_7_:Yb/Ho@KSc_2_F_7_. After naturally cooling down to room temperature, the obtained multilayer-structured nanorods were precipitated by the addition of ethanol, collected by centrifugation at 8000 rpm for 5 min, washed with ethanol several times, and finally re-dispersed in cyclohexane. By adjusting the orders and the Ln ion in the injected KSc_2_F_7_:Ln precursors, prepared as above, a series of KSc_2_F_7_ nanorods with distinct multilayer nanostructures can be readily obtained using similar synthesis procedures. It should be noted that the thickness of each layer in the multilayer-structured KSc_2_F_7_ nanorods can be tuned by changing the injected overall amount of corresponding Ln precursors.

## Results and discussion

In our design, orthorhombic-phase KSc_2_F_7_ was chosen as the host material for the synthesis of the color-tunable UC nanorods due to its ability to yield one-dimensional nanorods.^43^,^44^ Three typical sets of UC sensitizer/activator pairs of Yb^3+^/X^3+^ (X = Tm, Ho and Er) at precisely defined concentrations (ESI Fig. S2†) were separately incorporated into different layers of one single KSc_2_F_7_ nanorod to render the blue, green and red UC emissions *via* a modified stepwise oriented epitaxial growth method of thermal decomposition.^42^ Specifically, pure KSc_2_F_7_ interlayers with controllable thicknesses were intentionally fabricated to spatially separate the layers doped with three different Yb^3+^/X^3+^ pairs and thus minimize the energy loss induced by the unwanted CR-ETs (Scheme 1). As a result, monodisperse and uniform Ln^3+^-doped KSc_2_F_7_ nanorods with multilayer nanostructures like KSc_2_F_7_:Yb/Tm@KSc_2_F_7_@KSc_2_F_7_:Yb/Er@KSc_2_F_7_@KSc_2_F_7_:Yb/Ho@KSc_2_F_7_ (denoted as Tm@Er@Ho@Pure) were readily obtained in gram quantity (∼1.5 g) by a one-pot reaction with a yield as high as ∼93% based on the total molar quantities of the starting materials and injected KSc_2_F_7_:Ln precursors (ESI Fig. S3†). It should be noted that the outermost pure KSc_2_F_7_ layers were also fabricated to exclude the surface quenching effect of the UC emissions as is usually adopted in core–shell structured UCNPs. For comparison, KSc_2_F_7_:Yb/Tm@KSc_2_F_7_:Yb/Er@KSc_2_F_7_:Yb/Ho without pure KSc_2_F_7_ interlayers and Yb/Tm/Er/Ho co-doped KSc_2_F_7_ (denoted as Tm@Er@Ho and Tm/Er/Ho, respectively) nanorods were also synthesized using similar synthetic procedures.

**Fig. 1:**
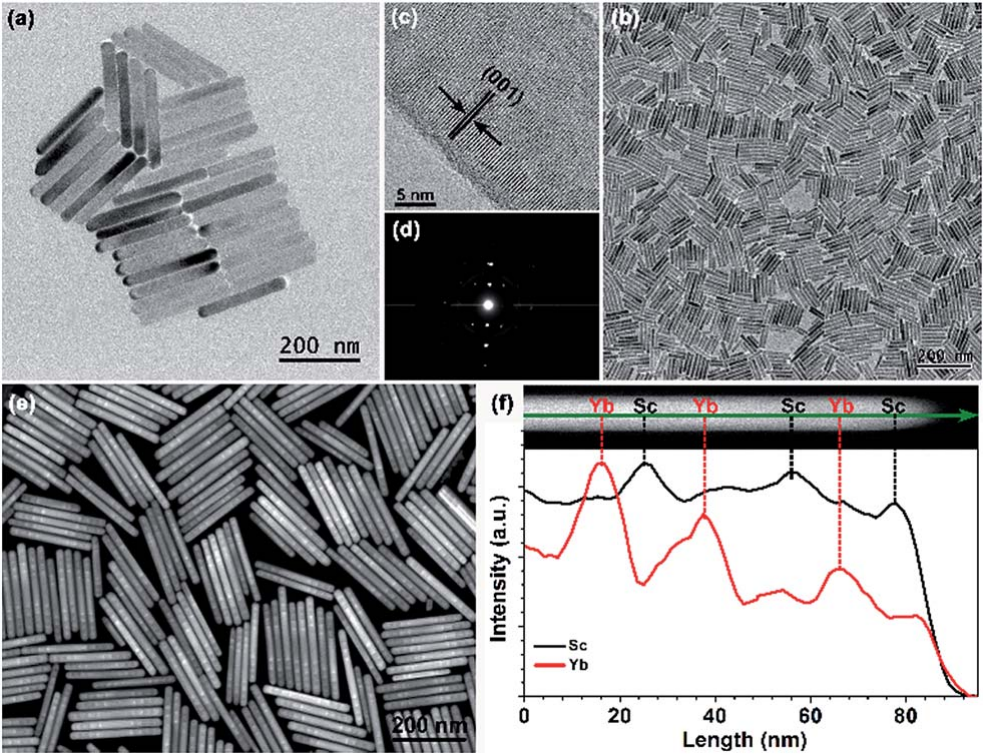
Typical low-resolution TEM images for (a) multilayer-structured KSc_2_F_7_ nanorods and (b) as-synthesized KSc_2_F_7_ seeds. (c) High-resolution TEM image of a single nanorod and its corresponding (d) selected-area electron diffraction pattern, revealing the single-crystalline nature of the multilayer-structured nanorods. (e) Representative HAADF-STEM image of the multilayer-structured KSc_2_F_7_ nanorods, demonstrating the contrast in density between the layers doped with Yb/X pairs and pure KSc_2_F_7_ interlayers. (f) EELS line scan conducted with HAADF-STEM imaging (upper) on half of a single nanorod, indicating the varied Sc and Yb content in different regions of the KSc_2_F_7_ nanorods that is in good consistency with the designed compositions of the multilayer nanostructure.

All the as-synthesized nanorods can be well indexed to be an orthorhombic-phase KSc_2_F_7_ crystal structure by XRD analysis (ESI Fig. S4†). Representative TEM images show that the as-synthesized segmented KSc_2_F_7_ nanorods have a long rod shape with an average length of 241 ± 12 nm and a width of 25 ± 3 nm (Fig. 1a), and are much longer than KSc_2_F_7_ seed nanorods of 150 ± 20 nm in length and 19 ± 3 nm in diameter (Fig. 1b), indicating the successful oriented epitaxial growth of the KSc_2_F_7_ nanorods. The corresponding high-resolution TEM image of an individual nanorod exhibits a clear lattice fringe with an observed *d*-spacing of 0.41 nm for the (001) plane of the orthorhombic KSc_2_F_7_ structure (Fig. 1c), revealing that the nanorods grow preferably along the [001] direction of the orthorhombic-phase KSc_2_F_7_, as further confirmed by the selected-area electron diffraction pattern of a randomly selected nanorod (Fig. 1d). To shed more light on the multilayer nanostructures, we conducted HAADF-STEM imaging of the as-synthesized nanorods (Fig. 1e). The discernible contrast in the pure KSc_2_F_7_ and Yb/X pair co-doped KSc_2_F_7_ layers associated with different atomic masses of Yb and Sc clearly demonstrates the formation of multilayer architectures. This finding can be further verified by the EELS analysis conducted on several randomly selected nanorods, in which the varied Sc and Yb content in different regions of the KSc_2_F_7_ nanorods is in accordance with the designed compositions of the multilayer nanostructures (Fig. 1f).

**Fig. 2:**
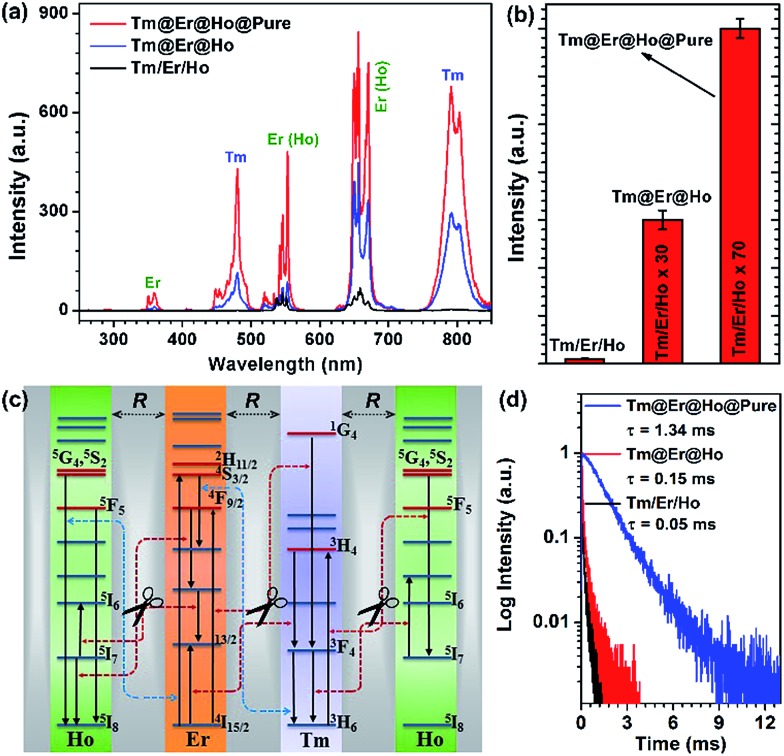
Comparison of (a) UC emission spectra and (b) their corresponding integrated intensities for the Tm/Er/Ho, Tm@Er@Ho and Tm@Er@Ho@Pure nanorods upon 980 nm excitation at a power density of 50 W cm^–2^. (c) Proposed mechanism for CR-ET inhibition among the Tm^3+^, Er^3+^ and Ho^3+^ ions in one single nanorod through fine-tuning the thickness (*R*) of the pure KSc_2_F_7_ interlayer. It should be noted that only partial energy levels and CR-ETs for the Tm, Er, and Ho ions are shown for clarity, and the dashed two end arrows represent the CR-ET processes. (d) UC luminescence decay curves of Tm emission centered at 792 nm upon 980 nm pulsed laser excitation in the Tm@Er@Ho@Pure (blue), Tm@Er@Ho (red) and Tm/Er/Ho (black) nanorods.

To demonstrate the feasibility of our design strategy, we first investigated the UC luminescence properties for the as-synthesized nanorods. As shown in Fig. 2a, upon 980 nm diode laser irradiation at a power density of 50 W cm^–2^, the Tm@Er@Ho@Pure nanorods display characteristic UC emission peaks centered at 480, 554, 650, 752 and 792 nm, which can be attributed to the intra-4f transitions of Tm^3+^, Er^3+^ (Ho^3+^), Er^3+^ (Ho^3+^), Tm^3+^ ions, respectively. In particular, the total integrated UC emission intensities for the Tm@Er@Ho@Pure and Tm@Er@Ho nanorods were found to be remarkably enhanced by a factor of about 70 and 30 as compared to the Tm/Er/Ho counterparts, respectively (Fig. 2b), which unambiguously reveals that the pure KSc_2_F_7_ interlayer plays a dominant role in enhancing the UC luminescence efficiency for these multilayer-structured KSc_2_F_7_ nanorods. Consistently, the absolute UC QYs were observed to increase markedly from 0.2% for the Tm/Er/Ho nanorods to 1.9% for the Tm@Er@Ho nanorods and to 3.9% for the Tm@Er@Ho@Pure nanorods upon 980 nm laser excitation at a power density of 50 W cm^–2^ (ESI Table S1†). To the best of our knowledge, such a high UC QY of 3.9% particularly for Yb/Tm/Er/Ho co-doped UCNPs has not yet been achieved before, and is found to be even higher than those of the well-established Yb/Er doped UC NaYF_4_ bulks or nanomaterials measured at the same laser excitation power density (ESI Table S1†).^45^ Likewise, such remarkably enhanced UC emissions were also achieved in the KSc_2_F_7_ nanorods doped with one or two pairs of Yb^3+^/X^3+^ (ESI Fig. S5 and S6†). Furthermore, we would like to emphasize that these multilayer-structured nanorods also outperform the mixture of three Yb/X-only doped KSc_2_F_7_ nanorods with almost identical lengths to the Tm@Er@Ho@Pure nanorods in terms of UC emission intensity and lifetime (ESI Fig. S7†), thereby clearly demonstrating the overwhelming advantages of the multilayer-structured design strategy over the conventional co-doping method in producing intense UC luminescence.

We attribute such highly enhanced UC properties to the successful inhibition of unwanted CR-ETs among Tm^3+^, Er^3+^ and Ho^3+^ ions across a large spatial distance resulting from the pure KSc_2_F_7_ interlayers of the Tm@Er@Ho@Pure nanorods which we intentionally fabricated. For the Tm/Er/Ho nanorods possessing a much smaller spatial distance in each dopant, the quenching of the UC luminescence is inevitable, due to the existence of severe CR-ETs among adjacent activators, leading to a much lower UC efficiency than those of the multilayer-structured nanorods. In contrast, those deleterious CR-ETs among Tm^3+^, Er^3+^ and Ho^3+^ ions such as ^5^G_4_, ^5^S_2_ → ^5^I_7_ (Ho):^3^H_6_ → ^3^H_4_ (Tm), ^5^G_4_, ^5^S_2_ → ^5^I_8_ (Ho):^4^I_15/2_ → ^4^S_3/2_ (Er), and ^1^G_4_ → ^3^F_4_ (Tm):^4^I_15/2_ → ^4^F_9/2_ (Er) can be effectively eliminated owing to the large activator-to-activator separation (*R*) isolated by the pure KSc_2_F_7_ interlayers within the multilayer-structured nanorods (Fig. 2c).^38^,^46^–^48^ Therefore, highly enhanced UC emissions with much better photo-stability were detected in the Tm@Er@Ho@Pure nanorods in comparison with their directly co-doped counterparts, as evidenced by the observation of easy burning in the directly co-doped nanorods upon 980 nm diode laser irradiation at a power density higher than ∼100 W cm^–2^ (ESI Fig. S8†).

**Fig. 3:**
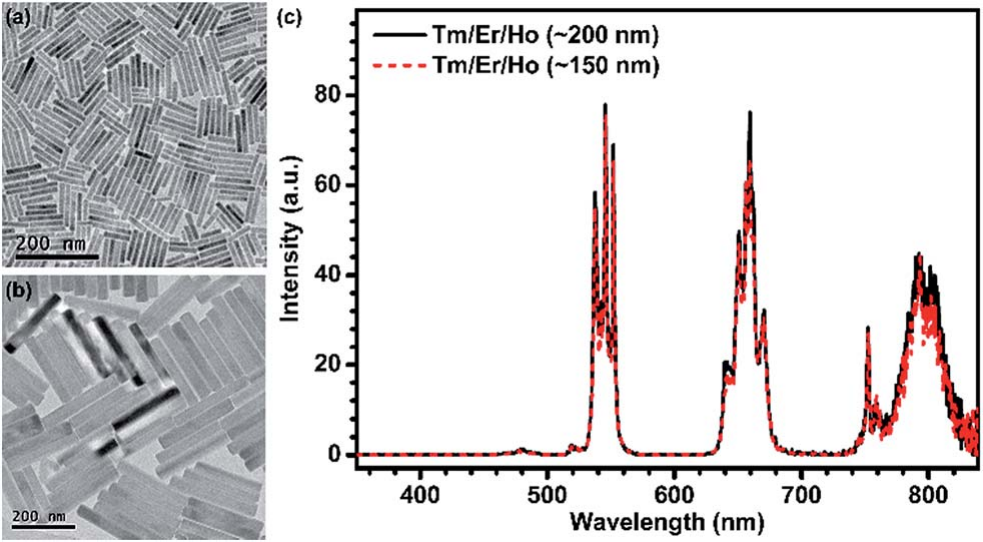
(a and b) TEM images of two types of Tm/Er/Ho nanorods with different lengths of ∼150 and ∼200 nm and diameters of ∼17 and ∼23 nm, and their corresponding (c) UC emission spectra upon 980 nm excitation at a power density of 50 W cm^–2^.

The successful inhibition of those unwanted CR-ETs can be further validated by UC luminescence decay studies. An increase in the Ln^3+^ UC lifetime is considered to be an explicit and convincing indicator for the suppression of CR-ETs, which certainly decreases the non-radiative relaxation rates of the excited energy levels of Ln^3+^ ions.^49^ As anticipated, all of the UC lifetimes for the Tm, Er and Ho ions in the Tm@Er@Ho@Pure nanorods were measured to be much longer than those in the Tm/Er/Ho and Tm@Er@Ho counterparts (ESI Fig. S9†). For instance, the mean UC lifetime for the ^3^H_4_ → ^3^H_6_ transition of Tm^3+^ in the Tm@Er@Ho@Pure nanorods was determined to be 1.34 ms, which is approximately 27 and 9 times longer than those in the Tm/Er/Ho (0.05 ms) and Tm@Er@Ho (0.15 ms) nanorods (Fig. 2d). To further verify the necessity of the pure KSc_2_F_7_ interlayer that separates different Yb^3+^/X^3+^ pairs from each other at an extremely large length scale for efficient UC luminescence, we synthesized the Tm/Er/Ho and the Yb/Er co-doped KSc_2_F_7_ nanorods with different lengths of ∼150 and ∼200 nm and diameters of ∼17 and ∼23 nm as the control (Fig. 3a and b and S10†). Both the UC emission intensity and the lifetime for the Tm/Er/Ho and the Yb/Er co-doped KSc_2_F_7_ nanorods were observed to remain essentially unchanged regardless of their varied nanorod lengths and diameters (Fig. 3c and S10 and S11†), which differs markedly from the core–shell structured UCNPs with size-dependent UC luminescence (ESI Fig. S12†), and thereby ruling out the possibility that the enhanced UC luminescence arises from the increased Ln^3+^ emitters with the increase in length and diameter of the nanorods. Taken together, these results provide clear evidence that the pure KSc_2_F_7_ interlayers in the KSc_2_F_7_ nanorods play a dominant role in eliminating the deleterious CR-ETs for highly enhanced UC luminescence.

**Fig. 4:**
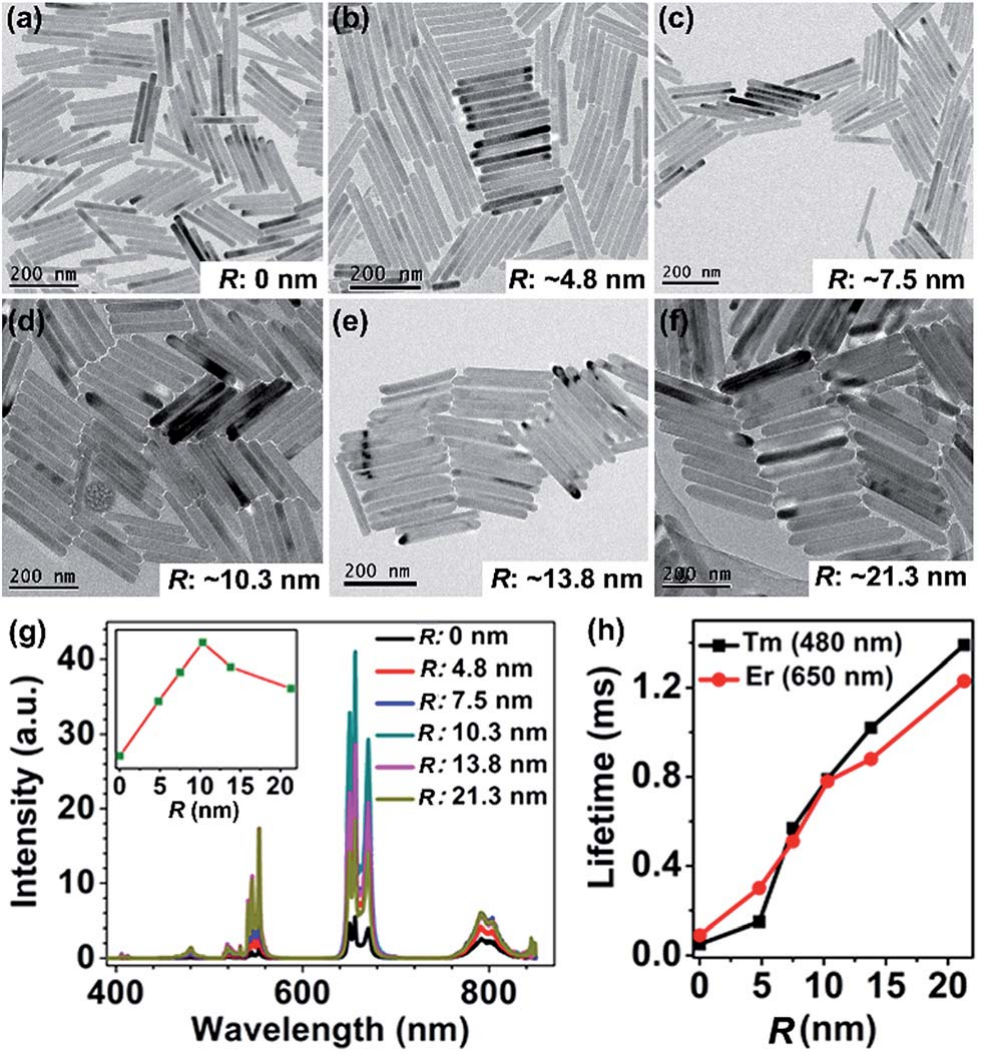
(a–f) TEM images for the Tm@Er@Pure nanorods with the pure KSc_2_F_7_ interlayer (*R*) ranging from 0 to ∼21.3 nm, and (g) their corresponding UC emission spectra. The inset of (g) shows the dependence of the integrated UC emission intensity on the thickness of the pure KSc_2_F_7_ interlayer. (h) UC lifetimes for the Er and Tm emissions centered at 650 and 480 nm as a function of the thickness of the pure KSc_2_F_7_ interlayer in the Tm@Er@Pure nanorods.

Under a dipole–dipole interaction, the probability (*W*_s–a_) for the CR-ETs between two given neighboring Ln^3+^ ions (provided that one Ln^3+^ ion acts as a sensitizer and the other one serves as an activator) is proportional to the inverse of the sixth power of their spatial distance (*R*_s–a_) and can be expressed as:^50^,^51^ 
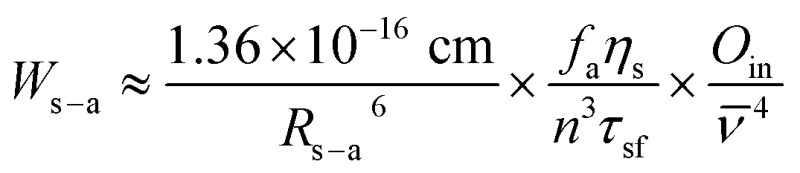
where *R*_s–a_, *n*, *f*_a_, *η*_s_ and *τ*_sf_ are the sensitizer–activator distance, refractive index, oscillator strength, quantum efficiency and the intrinsic luminescence lifetime of sensitizer; *ν̄* represents the average frequency in the overlap spectral region between the emission of the sensitizer and the absorption of the activator, and *O*_in_ is the overlap integral between the sensitizer emission and the activator absorption spectra. Therefore, we can deduce that the sensitizer–activator distance is one of the most critical parameters governing the CR-ETs for two given Ln^3+^ ions. To better understand the relationship between the UC luminescence and *R*_s–a_, we have synthesized a series of KSc_2_F_7_:Yb/Tm@KSc_2_F_7_@KSc_2_F_7_:Yb/Er@KSc_2_F_7_ and KSc_2_F_7_:Yb/Tm@KSc_2_F_7_@KSc_2_F_7_:Yb/Tm@KSc_2_F_7_ (hereafter referred to as Tm@Er@Pure and Tm@Tm@Pure, respectively) nanorods with a pure KSc_2_F_7_ interlayer thickness ranging from 0 to ∼21.3 nm and diameter from ∼8 to ∼15 nm (Fig. 4a–f and ESI Fig. S13 and 14†). The integrated UC emission intensities for both the Tm@Er@Pure and the Tm@Tm@Pure nanorods are observed to rise first with increasing the thickness of the pure KSc_2_F_7_ interlayer (0–10.3 nm) and then exhibit a gradual decrease upon further increasing the interlayer thickness (Fig. 4g and ESI Fig. S15†). In this sense, for a successful inhibition of CR-ETs, the optimum thickness of the pure interlayer is estimated to be ∼10.3 nm in the KSc_2_F_7_ nanorods. The initial enhancement in the UC emission intensity is conceivable due to the stepwise suppressed CR-ETs between the Er^3+^ and Tm^3+^ ions in view of the gradually increased interlayer thickness, whereas the subsequent decrease in the UC emission intensity is primarily associated with the reduced overall doping concentrations of the Tm and Er ions that deviate from their optimum values in the KSc_2_F_7_ nanorods owing to the excessive growth of the pure KSc_2_F_7_ interlayer. This explanation is strongly supported by the step-by-step prolonged UC lifetimes for both Er and Tm ions in the Tm@Er@Pure or Tm@Tm@Pure nanorods when the interlayer thickness increased from 0 to ∼21.3 nm (Fig. 4h and ESI Fig. S15 and S16†). To rule out the possibility that the enhanced UC emissions in the Tm@Er@Pure nanorods are primarily due to the outmost pure KSc_2_F_7_ protection layer, we compared the integrated UC emission intensities of the KSc_2_F_7_:Yb/Tm@KSc_2_F_7_:Yb/Er (without the pure KSc_2_F_7_ interlayer and outmost protection layer), KSc_2_F_7_:Yb/Tm@KSc_2_F_7_:Yb/Er@KSc_2_F_7_ (only with the outmost pure KSc_2_F_7_ protection layer of ∼10.3 nm) and Tm@Er@Pure nanorods with a pure KSc_2_F_7_ interlayer thickness of ∼10.3 and the outmost protection layer of ∼5 nm (ESI Fig. S17†). The UC luminescence intensity of the Tm@Er@Pure nanorods was determined to be 8.6 and 3.7 times stronger than those of the KSc_2_F_7_:Yb/Tm@KSc_2_F_7_:Yb/Er and KSc_2_F_7_:Yb/Tm@KSc_2_F_7_:Yb/Er@KSc_2_F_7_ nanorods, respectively, further demonstrating the dominant role of the pure KSc_2_F_7_ interlayer in improving the UC luminescence efficiency.

**Fig. 5:**
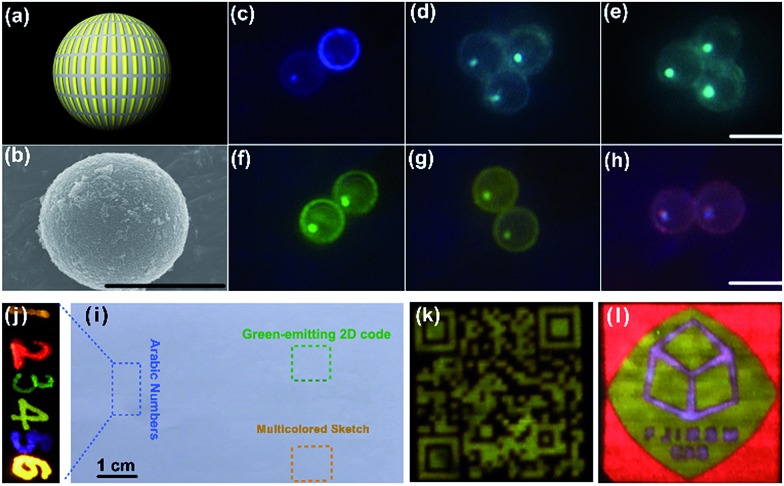
(a) Schematic illustration and (b) typical SEM image of polystyrene microbeads loaded with diverse multilayer-structured KSc_2_F_7_ nanorods, demonstrating the diversity of the UC color outputs from (c–h) blue to red under 980 nm diode laser irradiation (scale bar is 10 μm). UC photographs of the 2D code, multicolored sketch and Arabic numbers “123456” that were printed or handwritten on a piece of A4 paper using blue-, green-, yellow-, orange- and red-emitting multilayer-structured KSc_2_F_7_ nanorods as UC security ink in (i) daylight (or upon irradiation with a 365 nm UV lamp) and (j–l) upon 980 nm diode laser irradiation in the dark.

Based on the optimized interlayer thickness, we can fabricate a myriad of KSc_2_F_7_ nanorods doped with different Yb^3+^/X^3+^ pairs free of the negative effect of CR-ETs. By adjusting the Yb^3+^/X^3+^ doping combinations and locations (marked by a, b and c) in the multilayer-structured nanorods, overall 64 (that is, 4 × 4 × 4) kinds of KSc_2_F_7_ nanorods can be obtained with tunable UC emission outputs spanning from blue to red (ESI Fig. S18†), as indicated by the UC emission photographs of the polystyrene microbeads after loading with diverse multilayer-structured KSc_2_F_7_ nanorods (Fig. 5a–h and ESI Fig. S19†). Considering the diversity and flexibility of the multilayer nanostructures that we specially designed, these KSc_2_F_7_ nanorods may show great promise in applications as a new class of single-nanocrystal-based UC barcode for advanced anti-counterfeiting, which can greatly circumvent the deficiencies of those previously reported microcrystal-based UC barcodes utilizing merely two tips of NaYF_4_ microrods for encoding and thus are expected to be more difficult to replicate in practical anti-counterfeiting applications.^52^,^53^ As a proof-of-concept experiment, we printed a 2D code and a multicolored sketch, or handwrote the Arabic numbers of “123456” onto a piece of A4 paper using well-dispersed water-soluble blue-, green-, yellow-, orange- and red-emitting nanorods as UC security ink (ESI Fig. S20†). After naturally drying under ambient conditions, the patterns of the 2D code, sketch and Arabic numbers are almost invisible to the naked eye in daylight or upon irradiation with a 365 nm UV lamp (Fig. 5i). In contrast, all of them with blue to red UC emissions can be clearly distinguished on A4 paper when excited with a 980 nm NIR diode laser (Fig. 5j–l), thereby demonstrating the practicability of the multilayer-structured nanorods we developed to combat counterfeiting. As an added benefit, these nanorods can also be exploited as UC lifetime-tunable security ink by virtue of their varied UC decays from different Ln^3+^ ions embedded in the distinct layers of the KSc_2_F_7_ nanorods (ESI Fig. S21†).

## Conclusions

In summary, we have demonstrated a unique multilayer-structured strategy to remarkably enhance the UC luminescence of multiple Ln^3+^ ions embedded in one single nanorod, by manipulating the deleterious CR-ETs among diverse Ln^3+^ ions. The successful inhibition of the unwanted CR-ETs across the unusually large distance (>20 nm, a distance difficult to tune by the traditional core–shell strategy) allows us to fabricate a series of photo-stable UCNPs with an intensity more than 70 times higher than that of conventional UCNPs directly co-doped with Yb/X pairs. Benefiting from the diversity and flexibility of the multilayer nanostructures, these UCNPs exhibiting a wide range of UC emission color and lifetime outputs can function well as promising single-nanorod-based UC barcodes for advanced anti-counterfeiting. Such a strategy, paving a new way towards the precise control of CR-ETs that are ubiquitous in Ln^3+^-doped UCNPs, can be further generalized for the most promising UC luminescent system of β-NaYF_4_ (ESI Fig. S22†) and other lanthanide-doped confined systems such as 2D nanofilms or 0D nanodots, thus opening up a new avenue for the exploration of these multilayer-structured UCNPs for applications in displays, biosensing and anti-counterfeiting.

## Supplementary Material

Supplementary information
