# Supplementary material for: Manipulating energy transfer in lanthanide-doped single nanoparticles for highly enhanced upconverting luminescence
Source: Chem Sci. 2017 Apr 21;8(7):5050–6. doi: 10.1039/c7sc01393k (PMC5846170; doi:10.1039/c7sc01393k)
Supplement: Supplementary file 1 [file SC-008-C7SC01393K-s001.pdf]

Electronic Supplementary Material (ESI) for Chemical Science.
This journal is © The Royal Society of Chemistry 2017

Electronic Supplementary Information (ESI) for Chemical Science

Manipulating Energy Transfer in Lanthanide-Doped Single Nanoparticle for Highly Enhanced Upconverting Luminescence

Zhu Zhuo,[ab] Yongsheng Liu,[*a] Dajiu Liu,[a] Ping Huang,[a] Feilong Jiang,[a] Xueyuan Chen,[*a] and Maochun Hong[*a]

[a]CAS Key Laboratory of Design and Assembly of Functional Nanostructures, Key Laboratory of Optoelectronic Materials Chemistry and Physics, and State Key Laboratory of Structural Chemistry, Fujian Institute of Research on the Structure of Matter, Chinese Academy of Sciences, Fuzhou, Fujian 350002, China

[b]University of the Chinese Academy of Sciences, Beijing, 100049, China

*To whom correspondence should be addressed, e-mail: liuysh@fjirsm.ac.cn, hmc@fjirsm.ac.cn, and xchen@fjirsm.ac.cn

Supplementary Experimental Section

Chemicals and Materials. CF_3COOH, Ln_2O_3 (Ln = Sc, Y, Gd, Yb, Er, Ho and Tm), $Ln(CH_3COO)_3.4H_2O$, $CF_3COOK{\cdot}H_2O$, oleic acid (OA), oleylamine (OM), and 1-octadecence (ODE) were purchased from Sigma-Aldrich (China). $Ln(CF_3COO)_3.4H_2O$ was prepared as reported in the literature.[1] Polystyrene beads (9.0-9.9μm) were purchased from Aladdin Reagent Co., Ltd. 1-butanol, dichloromethane, cyclohexane, NaOH, NH_4F, and ethanol were purchased from Sinopharm Chemical Reagent Co., China. Unless otherwise noted, all the chemical reagents were used as received without further purification.

General procedure for the synthesis of Yb/X (X = Er, Tm and Ho) pair singly doped KSc_2F_7 nanorods with different lengths. Taking the KSc_2F_7:Yb20%/Er2% nanorods for example, 0.5 mmol of $CF_3COOK{\cdot}H_2O$, 0.78 mmol of $Sc(CF_3COO)_3{\cdot}4H_2O$, 0.2 mmol of $Yb(CF_3COO)_3{\cdot}4H_2O$ and 0.02 mmol of $Er(CF_3COO)_3{\cdot}4H_2O$ were first added to a 250 mL three-neck round-bottom flask containing 15 mL of OA, 10 mL of ODE and 5 mL of OM, and then heated at 150 °C under N_2 flow with constant stirring for 30 min to remove the water from the raw materials. The obtained mixture was heated to 300 °C under N_2 flow with constant stirring and maintained for 30 min to obtain ~150 nm KSc_2F_7:Yb/Er seed nanorods. After retrieving 1 mL of reaction mixture for TEM analysis, 10 mL KSc_2F_7:Yb/Er precursors were immediately injected into the reaction mixture and ripened at 300 °C for 30 min followed by the similar injection and ripening cycles. The length of the obtained KSc_2F_7:Yb/Er nanorods can be finely tuned by adjusting the injection and ripening cycles at 300 °C. After naturally cooling down to room temperature, the obtained KSc_2F_7:Yb/Er nanorods were

precipitated by addition of ethanol, collected by centrifugation at 8000 rpm for 5 min, washed with ethanol several times, and finally re-dispersed in cyclohexane.

General procedure for the synthesis of Yb/Er/Tm/Ho co-doped KSc_2F_7 nanorods with different lengths: In a typical procedure, 0.5 mmol of $CF_3COOK·H_2O$, 0.72 mmol of $Sc(CF_3COO)_3·4H_2O$, 0.2 mmol of $Yb(CF_3COO)_3·4H_2O$, 0.02 mmol of $Er(CF_3COO)_3·4H_2O$, 0.05 mmol of $Ho(CF_3COO)_3·4H_2O$, 0.01 mmol of $Tm(CF_3COO)_3·4H_2O$ were first added to a 250 mL three-neck round-bottom flask containing 15 mL of OA, 10 mL of ODE and 5 mL of OM, and then heated at 150 °C under N_2 flow with constant stirring for 30 min to remove the water from the raw materials. After retrieving 30 mL of reaction mixture as subsequent injected precursors, the remaining mixture was heated to 300 °C under N_2 flow with constant stirring and maintained for 30 min to obtain ~150 nm KSc_2F_7:Yb/Tm/Er/Ho nanorods. Thereafter, 10 mL KSc_2F_7:Yb/Tm/Er/Ho precursors were immediately injected into the reaction mixture and ripened at 300 °C for 30 min, followed by the similar injection and ripening cycles for several times. The length of the obtained KSc_2F_7:Yb/Tm/Er/Ho nanorods can be finely tuned by adjusting the injection and ripening cycles at 300 °C. After naturally cooling down to room temperature, the obtained KSc_2F_7:Yb/Tm/Er/Ho nanorods were precipitated by addition of ethanol, collected by centrifugation at 8000 rpm for 5 min, washed with ethanol several times, and finally re-dispersed in cyclohexane.

General procedure for the preparation of $NaGdF_4$:Yb/Er/Tm@$NaGdF_4$:Yb/Er/Tm core-shell nanoparticles (NPs). The synthesis of $NaGdF_4$:Yb/Er/Tm@$NaGdF_4$:Yb/Er/Tm core-shell NPs was conducted via a successive layer-by-layer injection protocol.[2] In a typical procedure, the $NaGdF_4$:Yb/Tm core NPs was first prepared by mixing 0.5 mmol of $Ln(CH_3COO)_3·4H_2O$ (Ln = Gd, Yb, Er and Tm), 4 mL OA and 8 mL ODE in a 100-mL three-neck round-bottom flask followed by heating at 150 °C for 30 min under N_2 flow. After cooling down to 50 °C, 5 mL of methanol solution containing NH_4F (2 mmol) and NaOH (1.25 mmol) was added and stirred for 30 min. Subsequently, the resulting mixture was heated to 290 °C under N_2 flow and maintained for 30 min to obtain ~10 nm $NaGdF_4$:Yb/Er/Tm core NPs. After retrieving 1mL of reaction mixture for TEM analysis, 2 mL ODE solution containing calculated amount of ~5 nm $NaGdF_4$:Yb/Er/Tm sacrificial shell NPs were immediately injected into the reaction mixture and ripened at 290 °C for 15 min followed by the similar injection and ripening cycles. The thickness of the $NaGdF_4$:Yb/Er/Tm shell layers can be finely tuned by adjusting the injection and ripening cycles. Injection of shell precursors and ripening cycles were performed to a total of three cycles to yield monodisperse ~15 nm $NaGdF_4$:Yb/Er/Tm@$NaGdF_4$:Yb/Er/Tm core-shell NPs. The obtained core-shell NPs were precipitated by addition of 30 mL of ethanol, collected by centrifugation, washed with ethanol several times, and finally re-dispersed in cyclohexane.

General procedure for the preparation of nanorod-tagged polystyrene beads. In a typical procedure, 100 μL of polystyrene beads (diameter 9-9.9 μm, 5% w/v) were first dispersed in the mixture of 1 mL 1-butanol and 1 mL dichloromethane, and then 500 μL dichloromethane solution containing 20 mg/mL multilayer-structured KSc_2F_7 nanorods was added and kept shaking gently at room temperature for 3 h. Thereafter, 4 mL of ethanol was added into the above mixture to stop the swelling of polystyrene beads, and the resulting nanorod-tagged polystyrene beads were collected by centrifugation at 6000 rpm for 5 min, washed with ethanol 3 times and redispersed in ethanol.

Preparation of water-soluble KSc_2F_7 nanorods. To render the hydrophobic nanorods hydrophilic, we removed the original OA ligands from their surface by acid treatment as previously reported.[2] In a typical process, 20 mg of the as-synthesized OA-capped KSc_2F_7 nanorods were dispersed in 30 mL of acidic ethanol solution (pH 1) and ultrasonicated for 30 min to remove the surface ligands. After the reaction, the nanorods were collected by centrifugation at 12000 rpm for 10 min, and further purified by adding an acidic ethanol solution (pH 4). The resulting water-soluble KSc_2F_7 nanorods were washed with ethanol and distilled water for several times, and then re-dispersed in distilled water for the following use.

Inkjet printing or handwritting of multilayer-structured KSc_2F_7 nanorods on banknote. A common ink cartridge of the commercial inkjet printer (HP Deskjet 1112) was washed with ethanol until the ink was completely cleared away. After the vacant cartridge was dried, 1 mL of water solution of water-soluble multilayer-structured KSc_2F_7 nanorods (30 mg/mL) was injected into the HP 803 cartridge. Subsequently, the printing was carried out by an inkjet printer connected with a computer, and thus the nanorods were patterned on a piece of A4 paper. The two-dimensional (2D) code and multicolored sketch were printed by using the inkjet printer, while the Arabic numbers of "123456" were handwritten on the A4 paper. The colorful UC luminescence pictures were taken by a Canon 70D camera upon irradiation using a 980-nm diode laser at a power density of ~200 W cm^{-2}, where a short pass filter of 750 nm was placed in the front of the camera to filter the 980-nm excitation light.

Theoretical Analysis for UC Luminescence Lifetime of Ln^{3+} Ion. It is well known that the observed lifetime (τ) for a particular excited state (luminescent level) of Ln^{3+} ion is determined by the sum of the inverse of the radiative and nonradiative rates, which can be expressed as: $\tau^{-1} = W_R + W_{NR}$, where W_R and W_{NR} are the total radiative and nonradiative relaxation rates from the excited state of interest, respectively.[3] The total radiative relaxation rate is mainly evaluated via Einstein spontaneous emission coefficients of Ln^{3+} ions, which can be regarded as a constant in a given host material. By contrast, the nonradiative relaxation rate is primarily determined by the magnitude of the energy gap between the luminescent and its next lowest-lying states of Ln^{3+} ions, which thereby can be significantly affected by

some factors such as surface defect states of NPs and CR-ETs between different or identical neighbouring Ln^{3+} ions that act as the nonradiative relaxation channels of excited states of Ln^{3+} ions. For the multilayer-structured KSc_2F_7 nanorods we obtained, the inhibition of CR-ETs between the adjacent Ln^{3+} ions through adjusting the thickness of pure KSc_2F_7 interlayer will certainly decreases the non-radiative relaxation rate (W_{NR}) of the excited states of Ln^{3+} ion and thus result in prolonged UC lifetimes. For the multilayer-structured KSc_2F_7 nanorods with pure KSc_2F_7 interlayer longer than 10.3 nm, the excessive growth of pure KSc_2F_7 interlayer will give rise to the decrease of the overall doping concentration of Ln^{3+} ions, which can also weaken the CR-ETs probability among Ln^{3+} ions, as observed in Yb/Tm co-doped $NaYF_4$ nanocrystals by Prof. Jin.[4] As a result, further prolonged UC lifetimes of Tm^{3+} and Er^{3+} ions were observed in Tm@Er@Pure or Tm@Tm@Pure nanorods with pure KSc_2F_7 interlayer longer than 10.3 nm.

Supplementary Figures

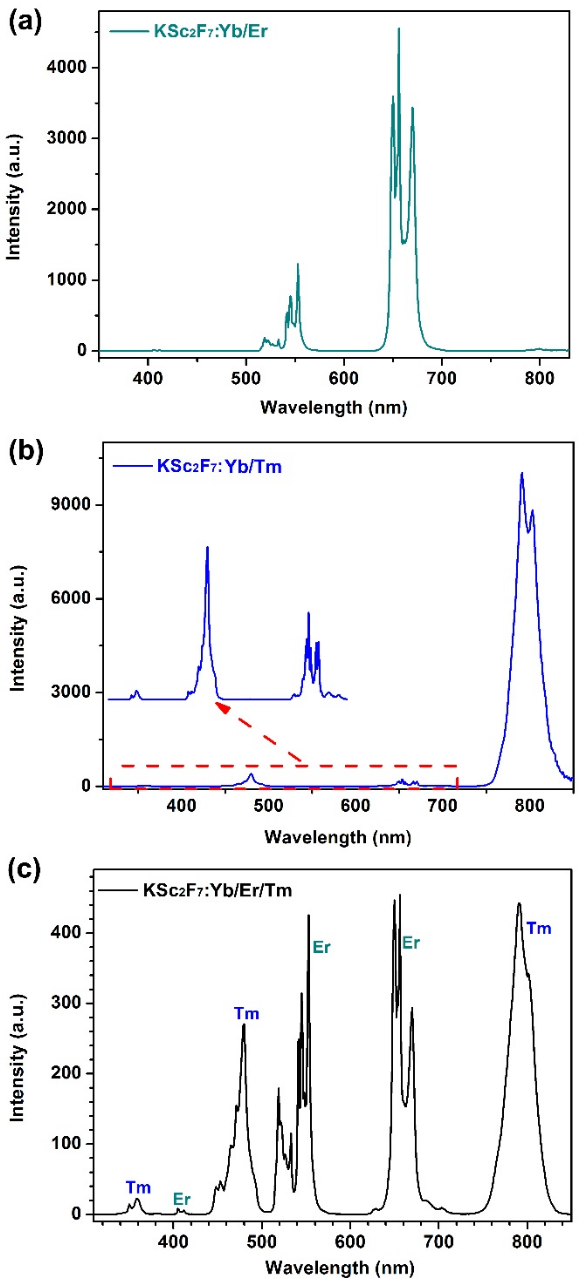

Fig. S1 Comparison of the UC luminescence intensities for Yb/Er, Yb/Tm and Yb/Er/Tm doped KSc_2F_7 nanorods, demonstrating the occurrence of the concentration quenching of UC luminescence in the case of multiple Ln ions co-doping. The doping concentration of Yb, Er and Tm were 20, 2 and 1 mol%, respectively. It can be clearly seen that the UC luminescence intensity for Yb/Er/Tm co-doped KSc_2F_7 nanorods is about one order of magnitude lower than those of Yb/Er or Yb/Tm pair singly doped KSc_2F_7 nanorods measured under identical experimental conditions, indicating the more severe concentration quenching for Yb/Er/Tm co-doped KSc_2F_7 nanorods with higher overall doping concentration of Er and Tm ions (3 mol%).

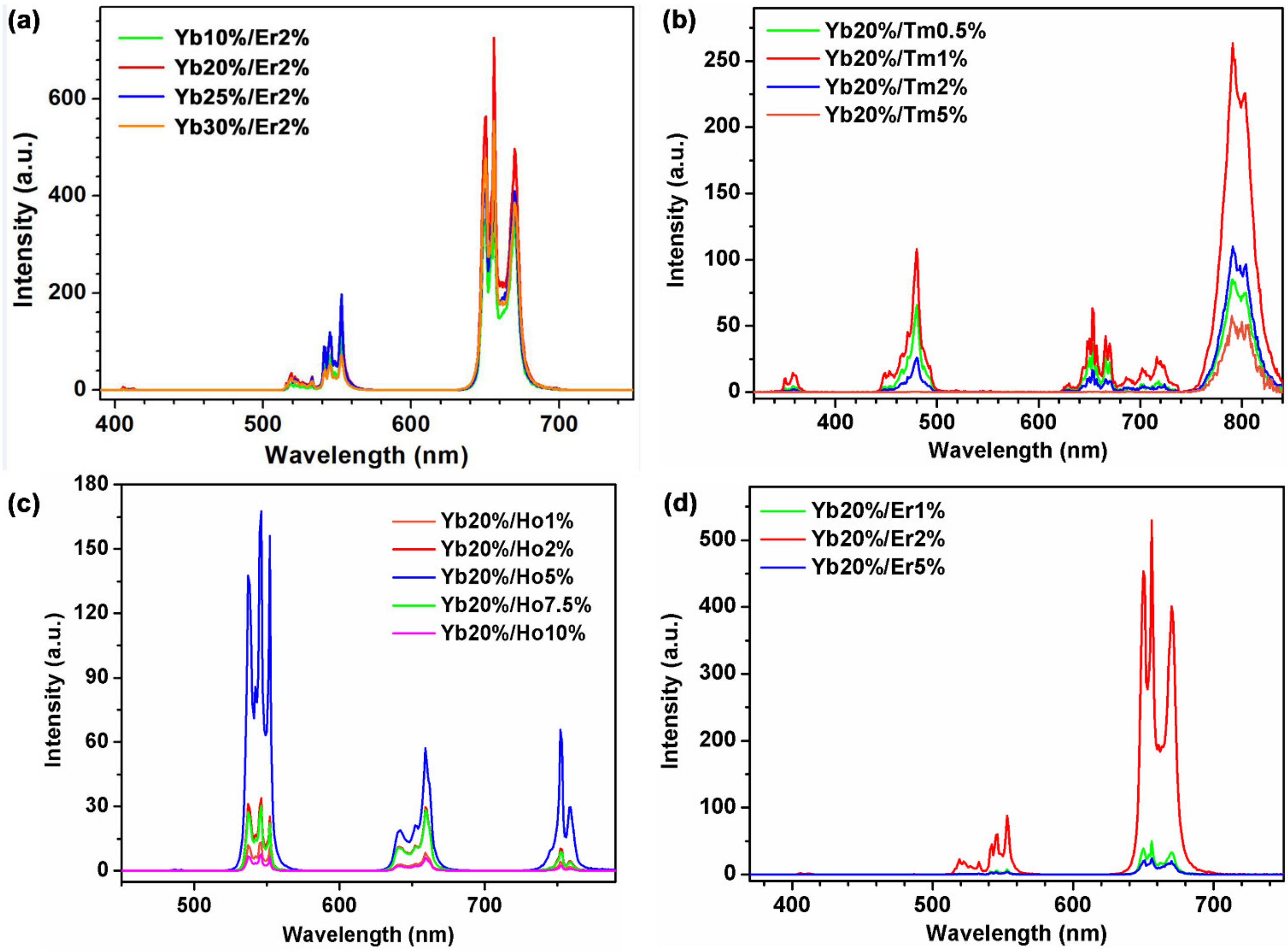

Fig. S2 UC emission spectra for a series of Yb/Er, Yb/Tm and Yb/Ho doped KSc_2F_7 nanorods as a function of different Yb, Er, Tm and Ho doping concentrations. All the UC emission spectra were measured under identical experimental conditions upon a 980-nm diode laser excitation with a power density of 50 W cm^{-2}. The results indicate that the optimum (a) Yb, (b) Tm, (c) Ho and (d) Er doping concentrations for intense UC luminescence in KSc_2F_7 nanorods were determined to be 20, 1, 5, and 2 mol%, respectively.

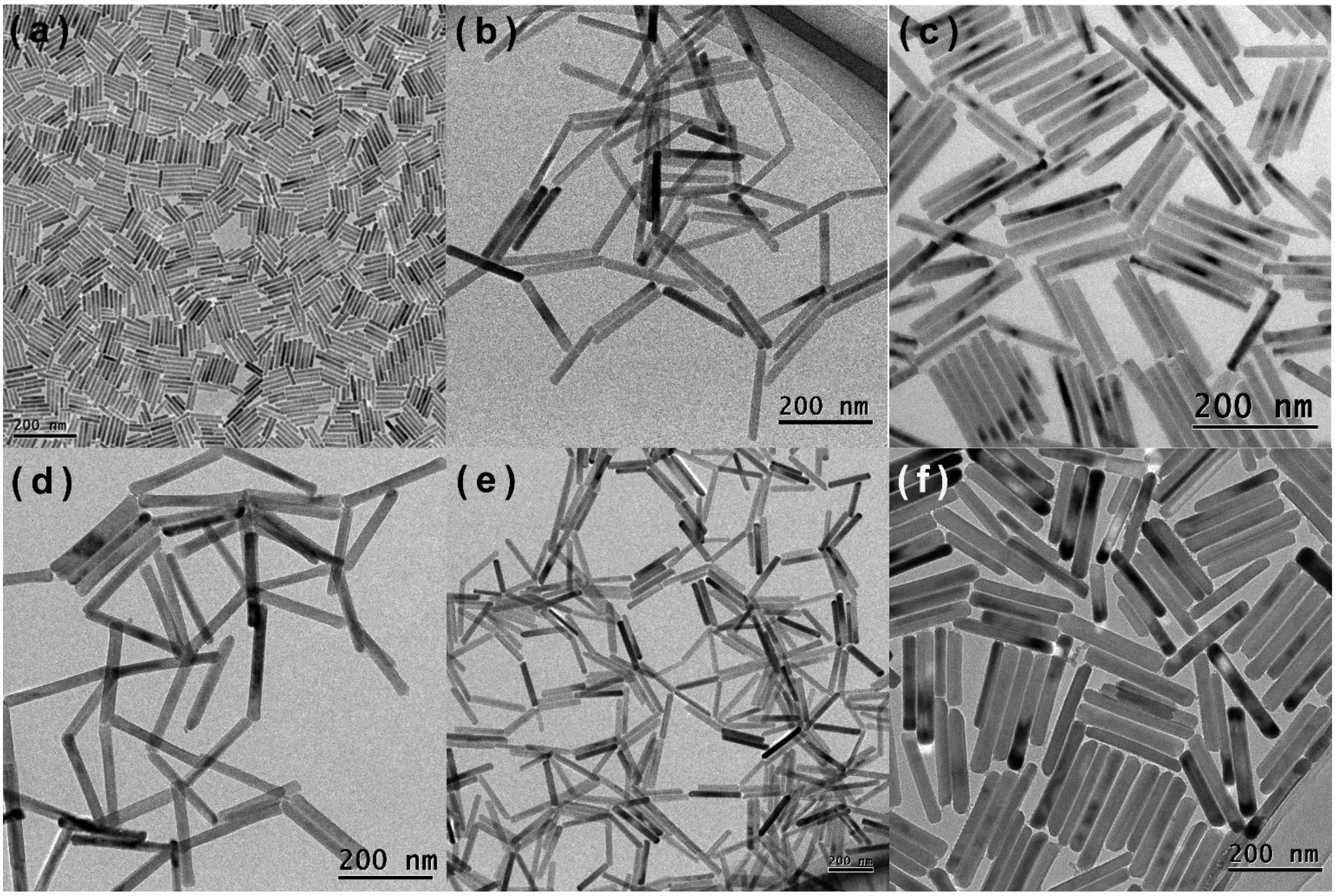

Fig. S3 Typical transmission electron microscopy (TEM) images for as-synthesized (a) KSc_2F_7:Yb/Tm seeds, (b) KSc_2F_7:Yb/Tm@KSc_2F_7, (c) KSc_2F_7:Yb/Tm@KSc_2F_7@KSc_2F_7:Yb/Er, (d) KSc_2F_7:Yb/Tm@KSc_2F_7@KSc_2F_7:Yb/Er@KSc_2F_7, (e) KSc_2F_7:Yb/Tm@KSc_2F_7@KSc_2F_7:Yb/Er@KSc_2F_7@KSc_2F_7:Yb/Ho, and (f) KSc_2F_7:Yb/Tm@KSc_2F_7@KSc_2F_7:Yb/Er@ KSc_2F_7@KSc_2F_7:Yb/Ho@ KSc_2F_7 nanorods retrieved at each stage of our synthesis procedures for Tm@Er@Ho@Pure nanorods, showing that all the samples have a rod shape with average lengths about 150, 170, 186, 210, 228 and 241 nm, respectively. The gradually increased nanorod lengths clearly demonstrate the successful oriented epitaxial growth and the formation of multilayer nanostructures of KSc_2F_7 nanorods.

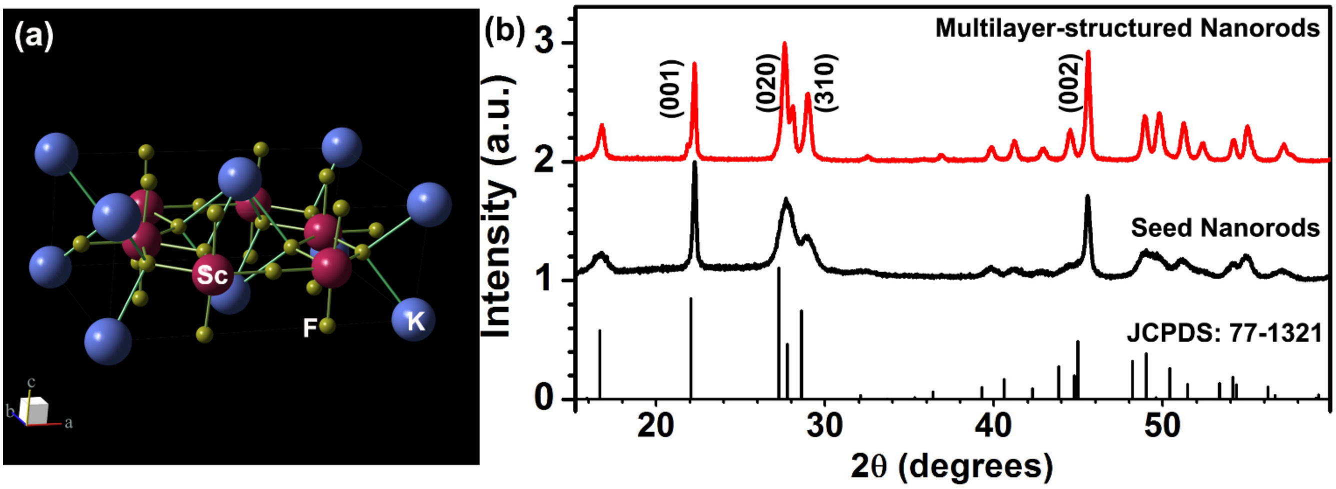

Fig. S4 (a) Schematic presentation of orthorhombic-phase KSc_2F_7 crystal structure. (b) XRD patterns for as-synthesized KSc_2F_7 seeds and multilayer-structured KSc_2F_7 nanorods, showing that all XRD peaks for seed and multilayer-structured KSc_2F_7 nanorods can be well indexed in accordance with orthorhombic-phase KSc_2F_7 crystal structure (JCPDS: No. 77-1321).

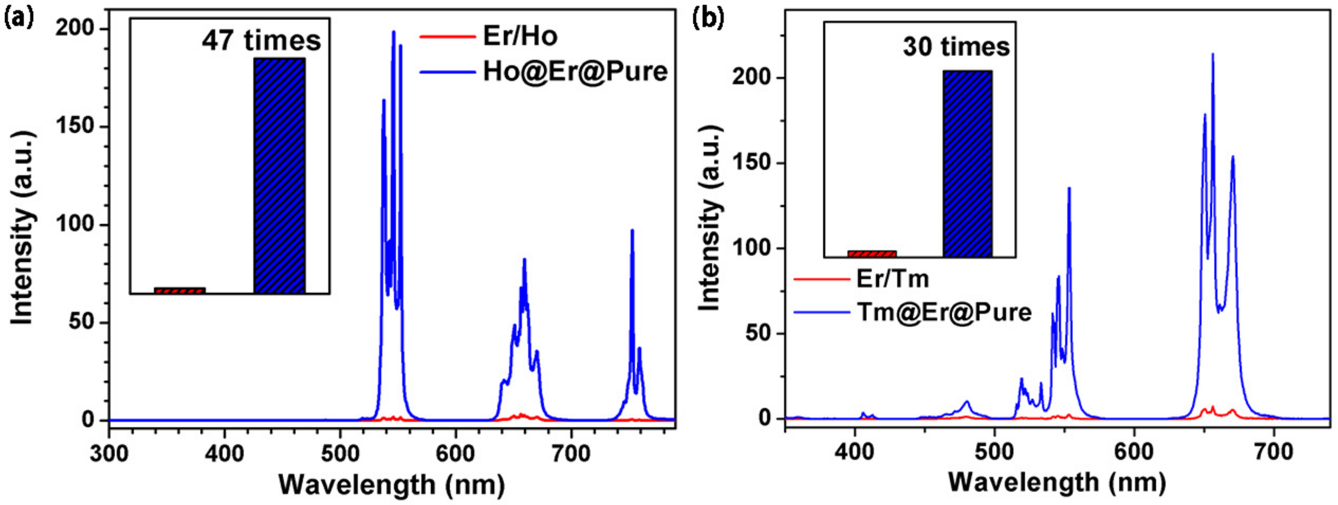

Fig. S5 Comparison of UC emission spectra and integrated UC emission intensities (inset) for (a) KSc_2F_7:Yb/Er/Ho (Er/Ho), KSc_2F_7:Yb/Ho@KSc_2F_7@KSc_2F_7:Yb/Er@KSc_2F_7 (Ho@Er@Pure) (b) KSc_2F_7:Yb/Er/Tm (Er/Tm) and KSc_2F_7:Yb/Tm@KSc_2F_7@KSc_2F_7:Yb/Er@KSc_2F_7 (Tm@Er@Pure) nanorods. All the UC emission spectra were measured under identical experimental conditions upon a 980-nm diode laser excitation with a power density of 50 W cm^{-2}. The integrated UC emission intensities for Ho@Er@Pure and Tm@Er@Pure nanorods were found to be enhanced by a factor of ~47 and ~30 when compared to their Er/Ho and Er/Tm counterparts, respectively, demonstrating the key role of pure KSc_2F_7 interlayer in enhancing the UC luminescence efficiency.

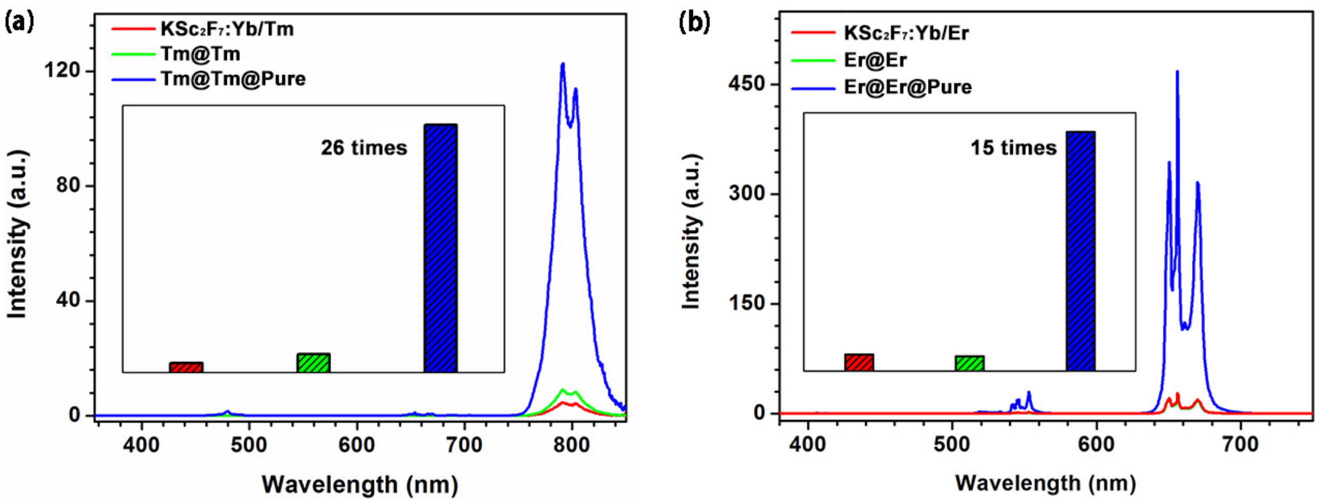

Fig. S6 Comparison of UC emission spectra and integrated UC emission intensities (Inset) for KSc_2F_7:Yb/Er, KSc_2F_7:Yb/Er@KSc_2F_7:Yb/Er (Er@Er), KSc_2F_7:Yb/Er@KSc_2F_7@KSc_2F_7:Yb/Er@KSc_2F_7 (Er@Er@Pure), KSc_2F_7:Yb/Tm, KSc_2F_7:Yb/Tm@KSc_2F_7:Yb/Tm (Tm@Tm), and KSc_2F_7:Yb/Tm@KSc_2F_7@KSc_2F_7:Yb/Tm@KSc_2F_7 (Tm@Tm@Pure) nanorods. All the UC emission spectra were measured under identical experimental conditions upon a 980-nm diode laser excitation with a power density of 50 W cm^{-2}. The integrated UC emission intensities for Er@Er@Pure and Tm@Tm@Pure nanorods were found to be 15-26 times higher than those of KSc_2F_7:Yb/Er, KSc_2F_7:Yb/Tm, Tm@Tm and Er@Er nanorods. These results clearly demonstrate that our design strategy works also very well for Yb/X pair singly doped KSc_2F_7 nanorods to suppress the unwanted CR-ETs occurred at the identical Ln ions, thereby revealing the versatility of our design strategy.

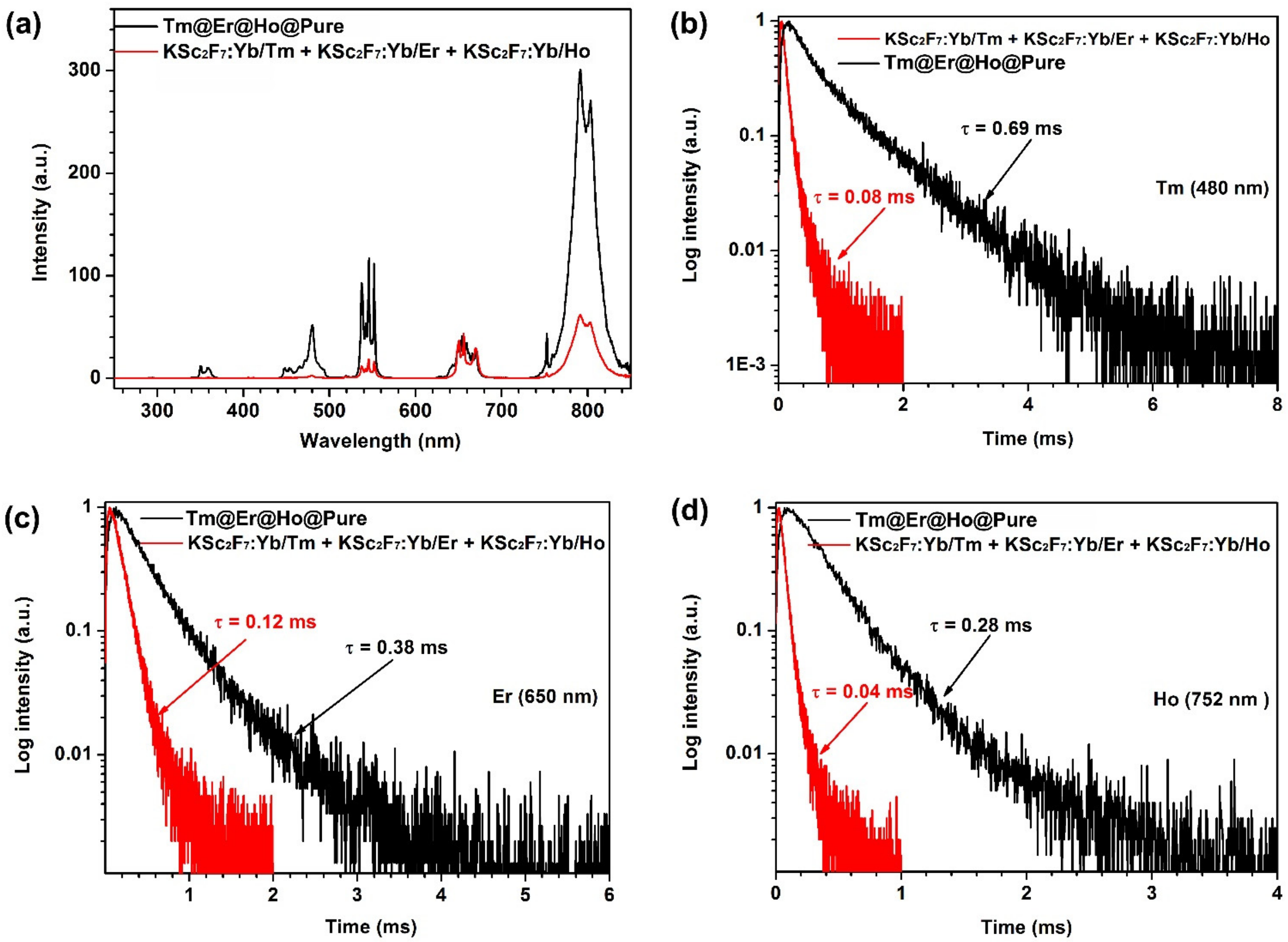

Fig. S7 Comparison of UC (a) luminescence spectra and (b, c and d) decay curves for Tm@Er@Ho@Pure nanorods and the mixture of KSc_2F_7:Yb/Er, KSc_2F_7:Yb/Tm and KSc_2F_7:Yb/Ho nanorods with almost identical length to the Tm@Er@Ho@Pure nanorods. The UC emission spectra were measured under identical experimental conditions upon a 980-nm diode laser excitation with a power density of 50 W cm^{-2}, while the UC luminescence decay curves were conducted by monitoring Tm, Er and Ho UC emissions centered at 480, 650 and 752 nm upon excitation by a 980-nm pulsed laser. The overall UC intensity for Tm@Er@Ho@Pure nanorods were observed to be 7 times higher than that of the mixture of the KSc_2F_7:Yb/Er, KSc_2F_7:Yb/Tm and KSc_2F_7:Yb/Ho nanorods with a mass ratio of 1:1:1, and the UC luminescence lifetimes for the Tm, Er and Ho ions in Tm@Er@Ho@Pure nanorods were also observed to be much longer than those of the mixture of the KSc_2F_7:Yb/Er, KSc_2F_7:Yb/Tm and KSc_2F_7:Yb/Ho nanorods. These results clearly demonstrate the advantages of multilayer-structured design strategy we proposed in producing intense UC luminescence, and thereby providing another solid evidence for the effective suppression of the unwanted CR-ETs in Tm@Er@Ho@Pure nanorods.

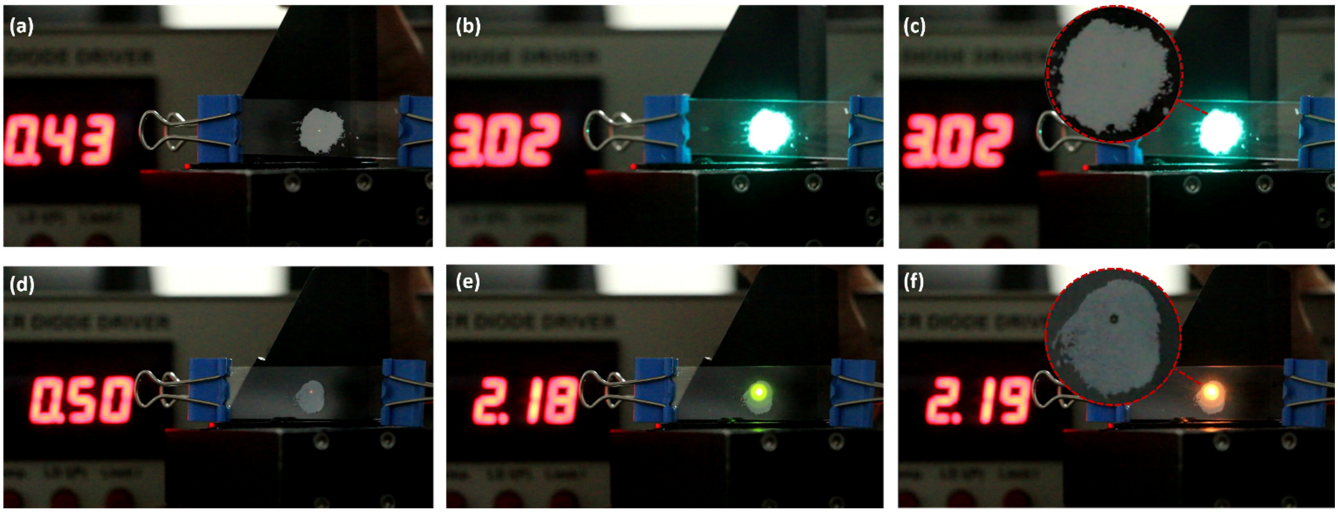

Fig. S8 UC emission photographs of (a-c) Tm@Er@Ho@Pure and (d-f) Yb/Tm/Er/Ho co-doped nanorods under 980-nm diode laser irradiation at different power densities, demonstrating the much better photo-stability (or thermal stability) of Tm@Er@Ho@Pure nanorods than that of directly co-doped nanorods. Note that the red values in Figure S6a-f represent the driving current of 980-nm diode laser instead of the power density, which were measured to be 4, 200, 200, 5, 100 and 101 W cm^{-2} by using a dynamometer. It can be clearly seen that the directly co-doped nanorods have been burnt out upon 980-nm diode laser at a power density of ~100 W cm^{-2}, while Tm@Er@Ho@Pure nanorods remain almost unchanged when excited at the power density even higher than ~ 200 W cm^{-2}. This phenomenon provides solid evidence for the existence of severe CR-ETs that can quench excitation energy and dissipate it non-radiatively as heat in Yb/Tm/Er/Ho co-doped nanorods, while such deleterious cross-relaxation type energy transfers can be effectively eliminated in Tm@Er@Ho@Pure nanorods and thus lead to much better photo-stability (or thermal stability) and UC emission efficiency in comparison with the co-doped nanorods.

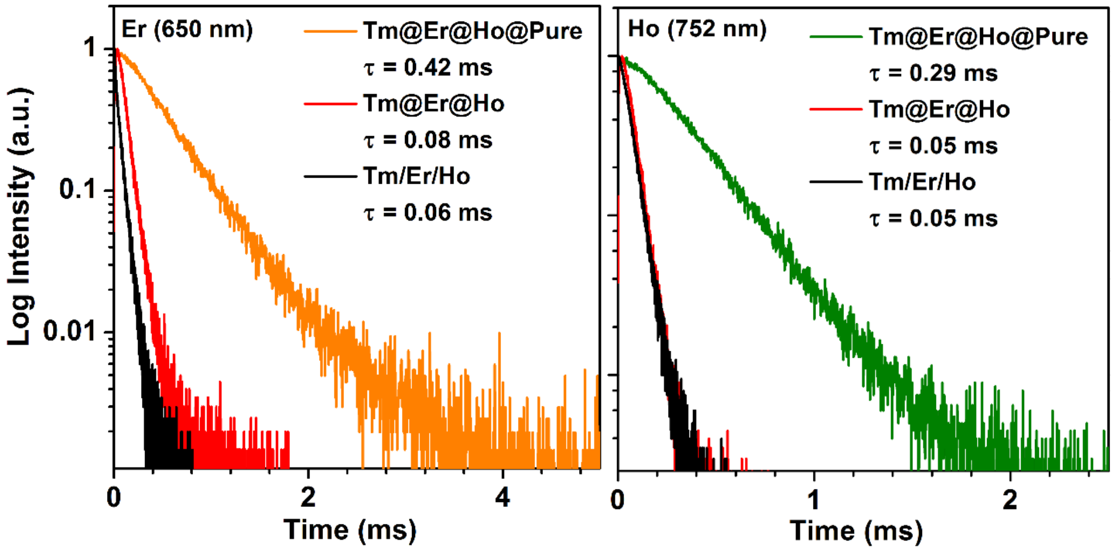

Fig. S9 UC luminescence decays of Er (left) and Ho (right) emissions centered at 650 and 752 nm in Tm@Er@Ho@Pure, Tm@Er@Ho and Tm/Er/Ho nanorods upon excitation at 980 nm. Both the UC lifetimes for Er and Ho ions in Tm@Er@Ho@Pure nanorods were measured to be much longer than those in Tm/Er/Ho (or Tm@Er@Ho) counterparts, showing that the unwanted CR-ETs can be effectively eliminated in Tm@Er@Ho@Pure nanorods and thereby resulting in much prolonged UC lifetimes of Er and Ho ions when compared to Tm@Er@Ho and Tm/Er/Ho nanorods.

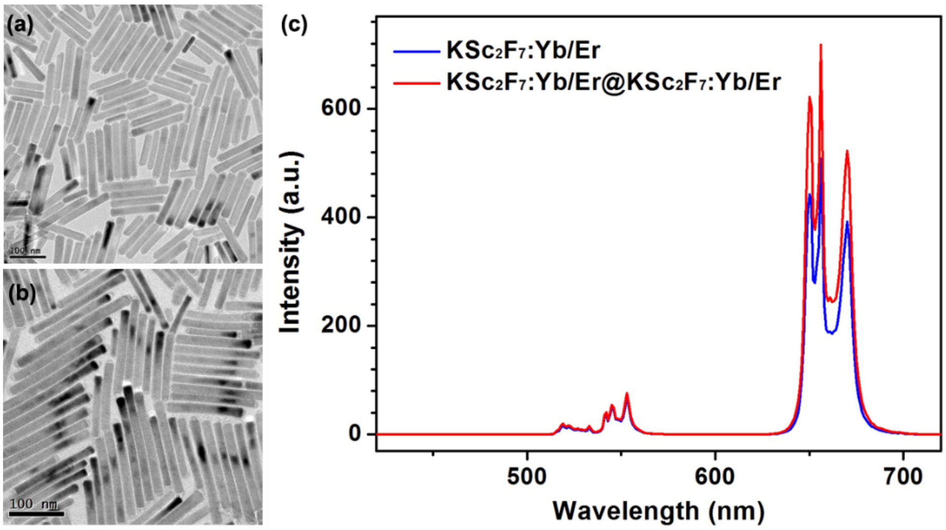

Fig. S10 (a, b) TEM images of two types of Yb/Er co-doped KSc_2F_7 nanorods with different lengths of ~150 and ~200 nm and diameters of ~17 and ~23 nm, and their corresponding (c) UC emission spectra upon 980-nm excitation at a power density of 50 W cm^{-2}. It was found that the integrated UC emission intensity for Yb/Er co-doped KSc_2F_7 nanorods were observed to remain almost unchanged regardless of their varied nanorod lengths and diameters, which thereby rules out the possibility that the enhanced UC luminescence arises from the increased Ln^{3+} emitters when the length and diameter of multilayer-stuctured KSc_2F_7 nanorods are increased.

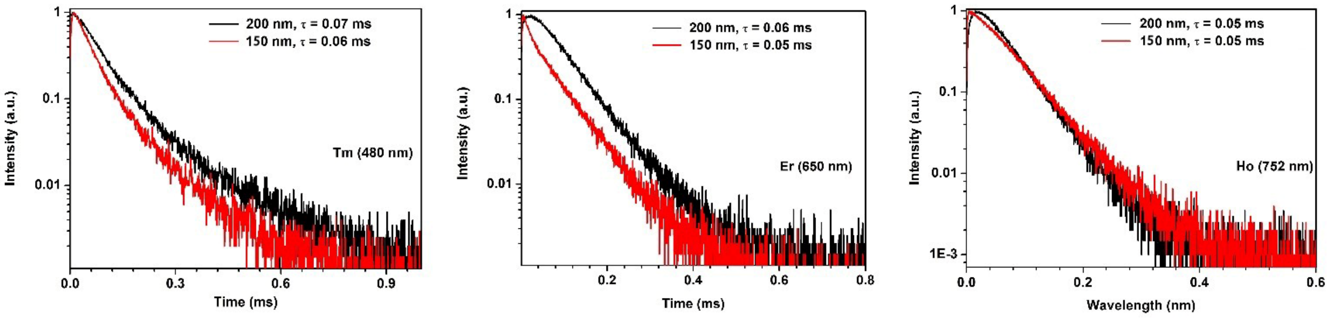

Fig. S11 Comparison of UC luminescence decays for Yb/Tm/Er/Ho co-doped KSc_2F_7 nanorods with lengths of ~150 nm and ~200 nm by monitoring Tm, Er and Ho UC emissions centered at 480, 650 and 752 nm upon excitation using a 980-nm pulsed laser. All the UC luminescence lifetimes for the Tm, Er and Ho ions were observed to be almost unchanged with the varied nanorod lengths. This phenomenon is understandable due to the almost identical surface-to-volume ratios of Tm, Er and Ho ions in the Yb/Tm/Er/Ho co-doped KSc_2F_7 nanorods regardless of their varied nanorod lengths.

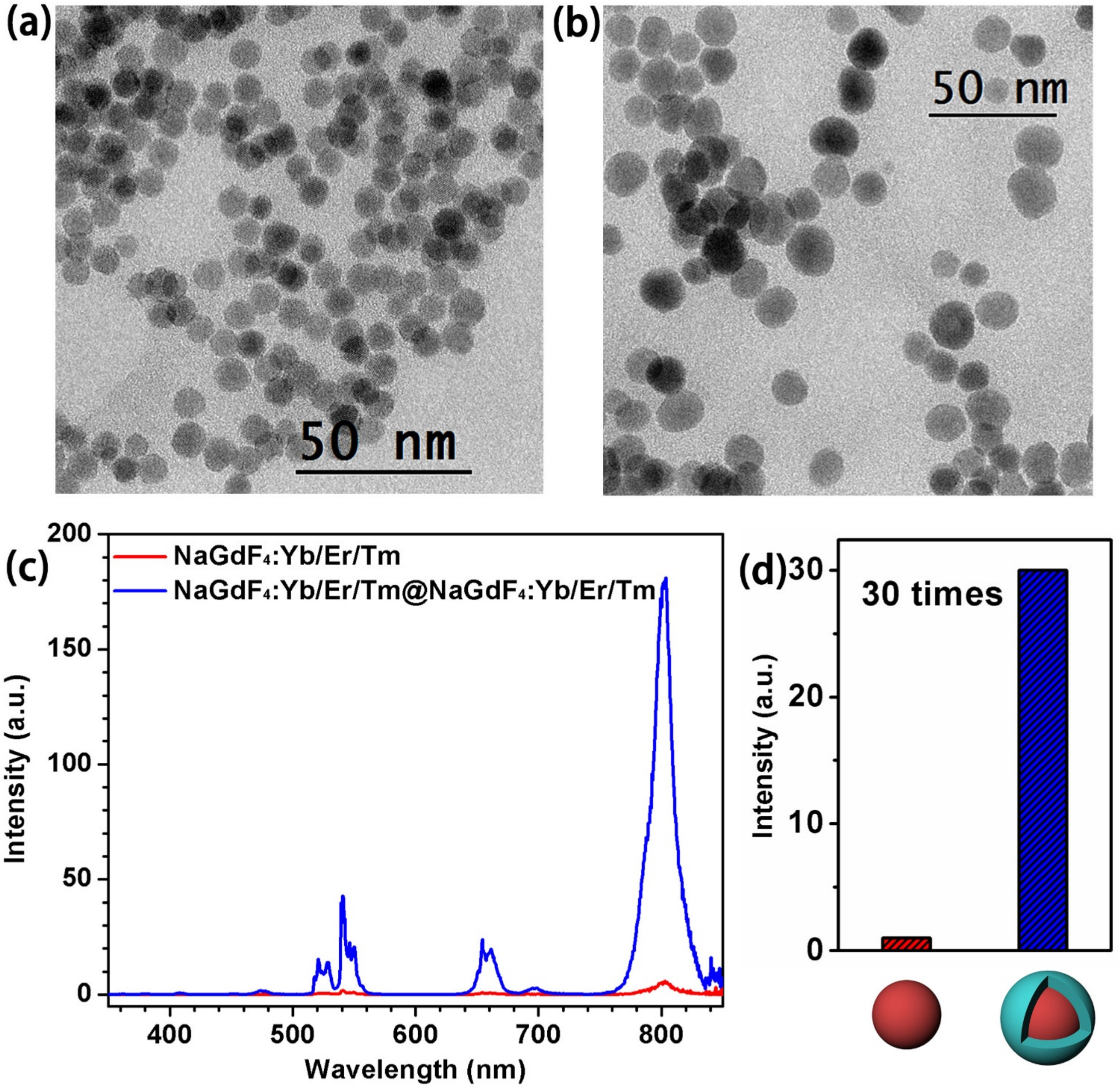

Fig. S12 TEM images for hexagonal (a) $NaGdF_4$:Yb/Er/Tm core and (b) $NaGdF_4$:Yb/Er/Tm@$NaGdF_4$:Yb/Er/Tm core-shell NPs with an average nanoparticle size ~10 and ~15 nm, and their corresponding UC (c) luminescence spectra and (d) integrated intensities. The UC intensity for core-shell NPs was observed to be enhanced by a factor of ~30 times relative to the core NPs when the nanoparticle size increased from ~10 to ~15 nm. This result clearly demonstrates that the enhanced UC luminescence for core-shell NPs is associated with the inhibition of surface quenching effect rather than the unwanted CR-ETs between Er and Tm ions, in view of the fact that Er and Tm ions are uniformly distributed in both the core and core-shell NPs.

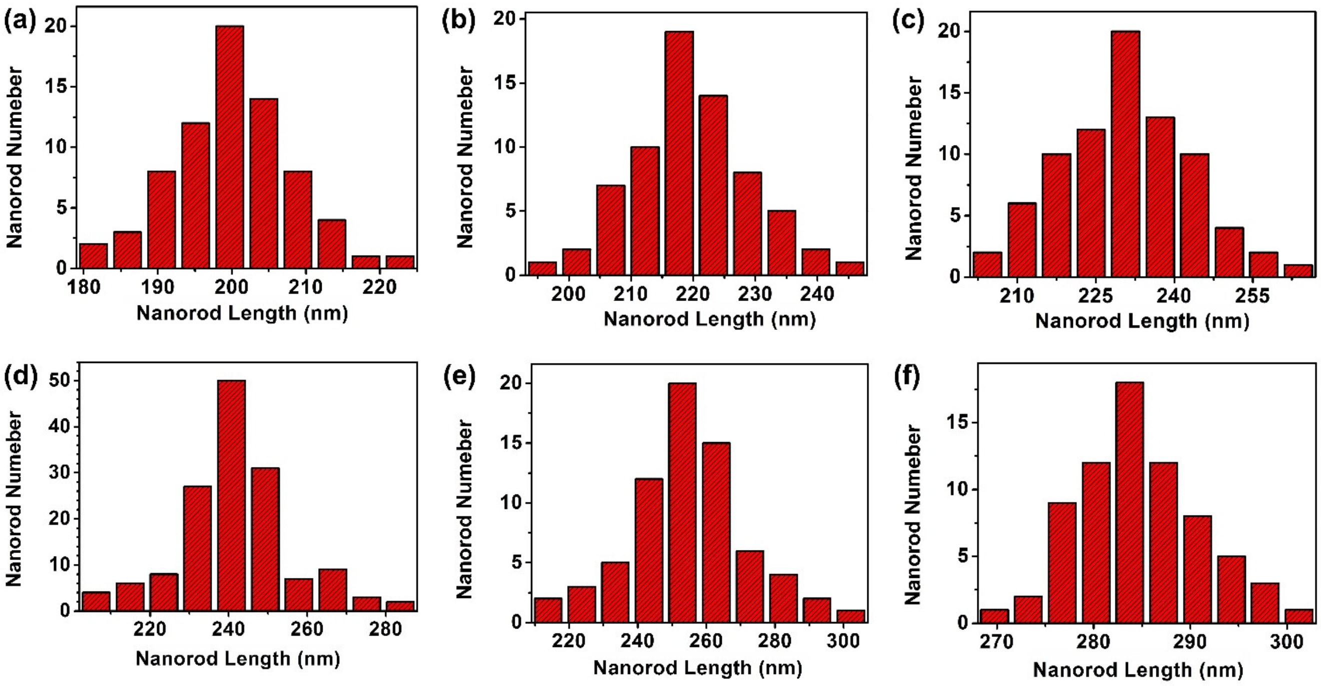

Fig. S13 Size distribution of a series of KSc_2F_7:Yb/Tm@KSc_2F_7@KSc_2F_7:Yb/Er@KSc_2F_7 (Tm@Er@Pure) nanorods with different thicknesses of pure KSc_2F_7 interlayer. The average lengths for the Tm@Er@Pure nanorods were determined to be 200 ± 9, 219 ± 8, 230 ± 12, 241 ± 6, 255 ± 8 and 285 ± 12 nm on the basis of the length histograms obtained from TEM images of 150 nanorods. Accordingly, the thickness of pure KSc_2F_7 interlayers were estimated to be 0, ~4.8, ~7.5, ~10.3, ~13.8 and ~21.3 nm, respectively, based on the amount of pure KSc_2F_7 interlayer precursors injected in our synthesis procedures.

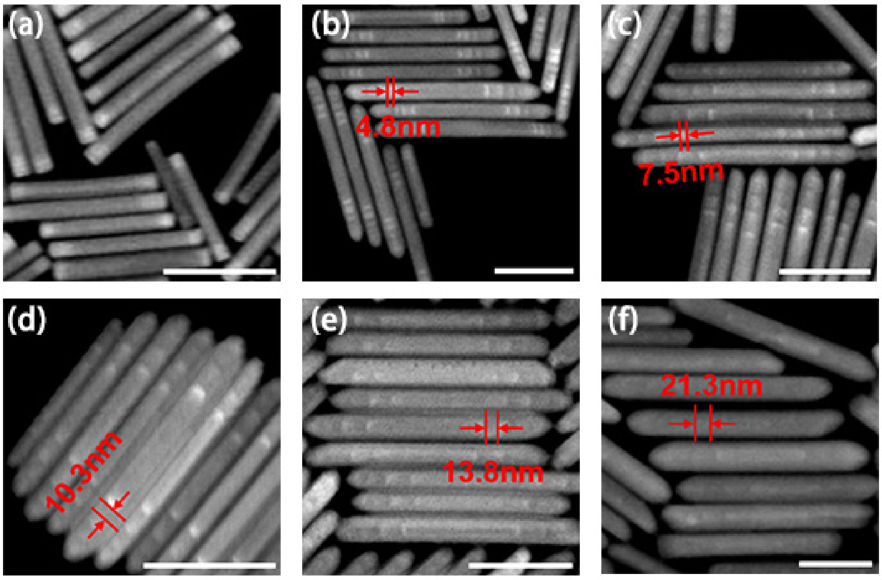

Fig. S14 Representative high angle annular dark-field scanning TEM (HAADF-STEM) images for Tm@Er@Pure nanorods with varied thicknesses of pure KSc_2F_7 interlayer. The discernible contrast for pure KSc_2F_7 and Yb/Er (or Yb/Tm) pair doped KSc_2F_7 layers in HAADF-STEM images clearly demonstrates the formation of multilayer architectures of Tm@Er@pure nanorods with varied thicknesses of pure KSc_2F_7 interlayer from 0 to 21.3 nm and cross section radius from about 8 to 15 nm (scale bar = 100 nm).

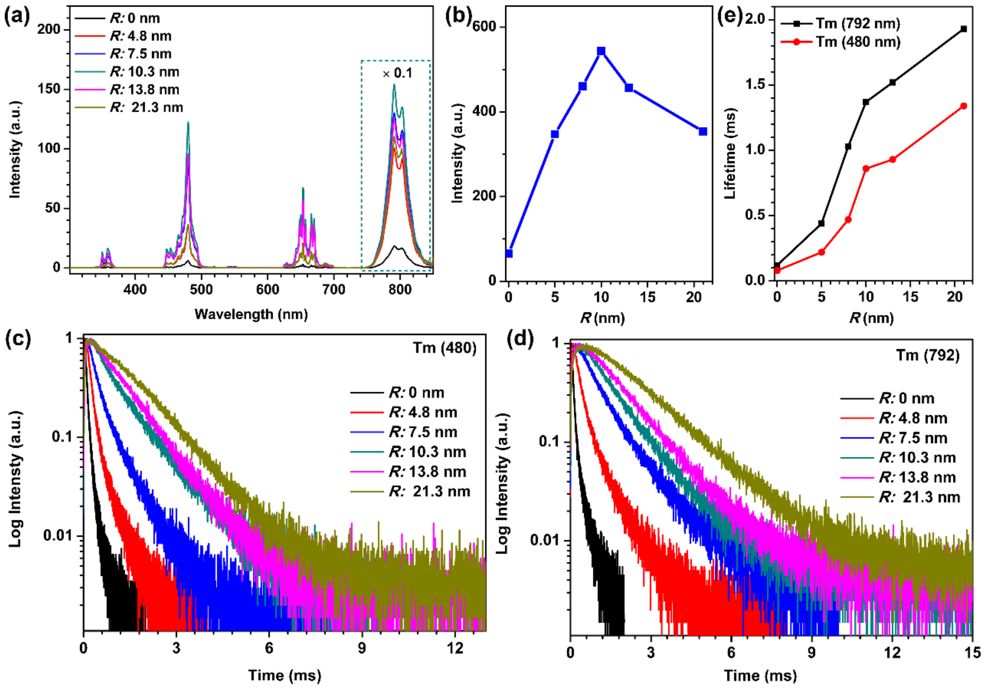

Fig. S15 (a) Comparison of UC luminescence spectra for KSc_2F_7:Yb/Tm@KSc_2F_7@KSc_2F_7:Yb/Tm@KSc_2F_7 (Tm@Tm@Pure) nanorods with different thicknesses of pure KSc_2F_7 interlayers ranging from 0 to ~21.3 nm, and (b) their corresponding integrated UC emission intensity on the thickness of pure KSc_2F_7 interlayer. UC luminescence decays from (c) $^1G_4$ and (d) $^3H_4$ states of Tm ion in Tm@Tm@Pure nanorods by monitoring Tm UC emissions centered at 480 and 792 nm upon excitation by a 980-nm pulsed laser, and e) their corresponding UC lifetime as a function of the thickness of pure KSc_2F_7 interlayer. Similar to the case of Tm@Er@Pure nanorods, the integrated UC emission intensity for Tm@Tm@Pure nanorods is also observed to rise at first and then decrease afterwards with increasing thickness of pure KSc_2F_7 interlayer from 0 to ~21.3 nm. The initial enhancement in UC emission intensity is conceivable due to the stepwise suppressed CR-ETs between Er^{3+} and Tm^{3+} ions in view of the gradually increased interlayer thickness, whereas the subsequent decrease in UC emission intensity is primarily associated with the reduced overall Tm doping concentration that deviates from their optimum ones in KSc_2F_7 nanorods owing to the excessive growth of pure KSc_2F_7 interlayer. As a result, step-by-step prolonged UC lifetimes of $^1G_4$ (480 nm) and $^3H_4$ (792 nm) states of Tm ion are detected in Tm@Tm@Pure nanorods, due to the fact that both the stepwise suppressed CR-ETs and reduced overall doping concentration of Tm ion will certainly decrease the non-radiative relaxation rates for the excited energy levels of Tm^{3+} ion and thus result in the prolonged UC lifetimes.

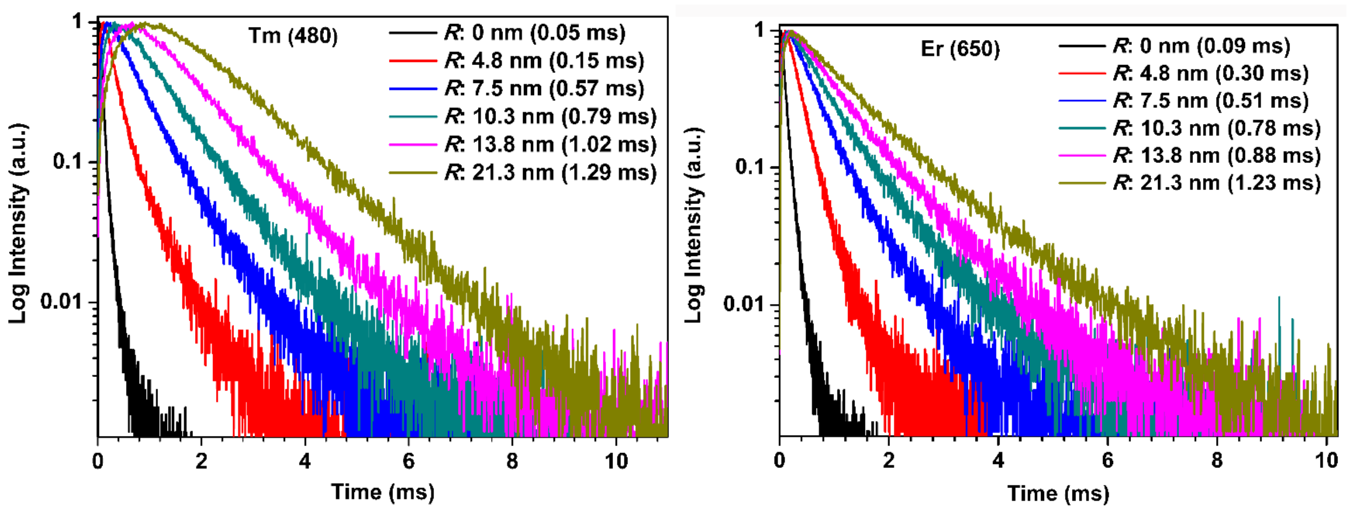

Fig. S16 Comparison of UC luminescence decays for KSc_2F_7:Yb/Tm@KSc_2F_7@KSc_2F_7:Yb/Er@KSc_2F_7 (Tm@Er@Pure) nanorods with different thicknesses of pure KSc_2F_7 interlayers ranging from 0 to ~21.3 nm, by monitoring Tm (left) and Er (right) UC emissions centered at 480 and 650 nm upon excitation using a 980-nm pulsed laser. It can be clearly seen that the UC lifetimes for both Er and Tm ions in Tm@Er@Pure nanorods increase gradually with the increased thickness of pure KSc_2F_7 interlayer. This phenomenon is expected in view of the fact that both the suppressed CR-ETs and/or reduced overall doping concentrations of Tm and Er ions in Tm@Er@Pure nanorods by tuning the thickness of pure KSc_2F_7 interlayer will certainly decrease the non-radiative relaxation rates for the excited energy levels of Tm and Er ions, thus giving rise to the prolonged UC lifetimes.

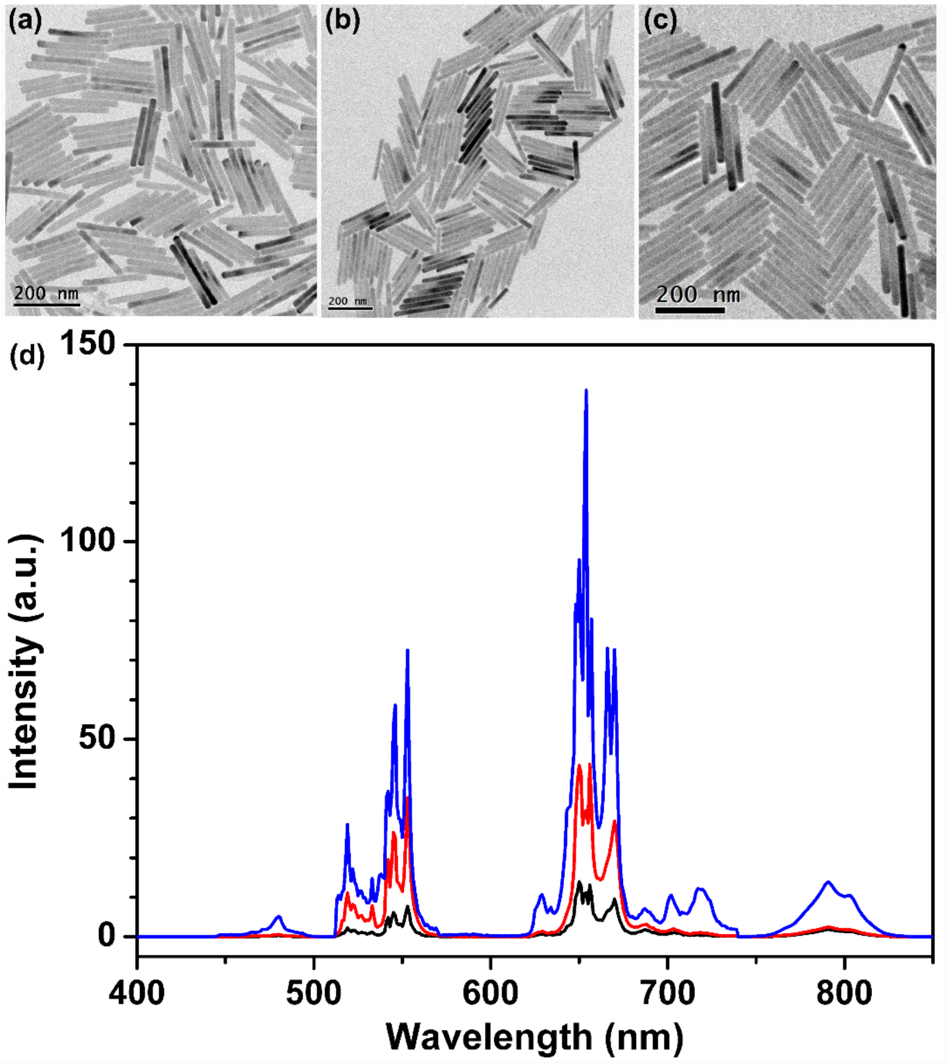

Fig. S17 (a-c) TEM images for KSc_2F_7:Yb/Tm@KSc_2F_7:Yb/Er (without pure KSc_2F_7 interlayer and outmost protection layer), KSc_2F_7:Yb/Tm@KSc_2F_7:Yb/Er@KSc_2F_7 (only with the outmost KSc_2F_7 protection layer of ~10.3 nm) and KSc_2F_7:Yb/Tm@KSc_2F_7@KSc_2F_7:Yb/Er@KSc_2F_7 (with the outmost protection layer of ~5 nm and pure KSc_2F_7 interlayer of ~10.3 nm) nanorods with increased cross section radii of ~8, ~9 and ~10 nm, respectively, and (d) their corresponding UC emission spectra upon 980-nm excitation at a power density of 50 W cm^{-2}. The UC luminescence intensity for KSc_2F_7:Yb/Tm@KSc_2F_7@KSc_2F_7:Yb/Er@KSc_2F_7 (blue) nanorods was determined to be 8.6 and 3.7 times stronger than those KSc_2F_7:Yb/Tm@KSc_2F_7:Yb/Er (black) and KSc_2F_7:Yb/Tm@KSc_2F_7:Yb/Er@KSc_2F_7 (red) nanorods, respectively, which unambiguously suggests that the enhanced UC emissions for Tm@Er@Pure nanorods are primarily originated from the pure KSc_2F_7 interlayer instead of the outmost KSc_2F_7 protection layer.

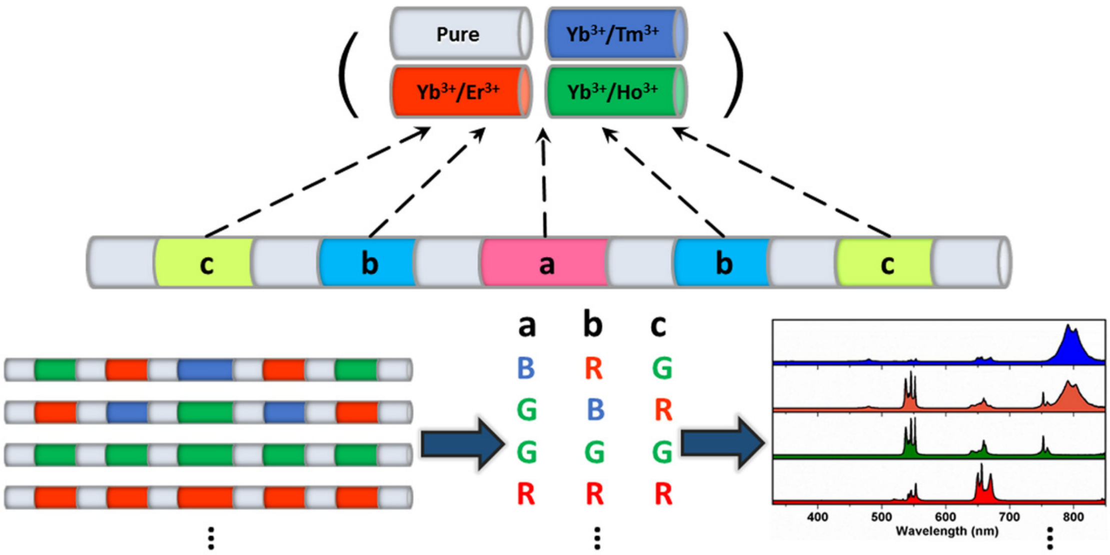

Fig. S18 Schematic design of multilayer-structured KSc_2F_7 nanorod comprising Yb/Tm, Yb/Er and Yb/Ho pairs separately incorporated layers and pure KSc_2F_7 interlayers to render the red, green and blue UC emissions in one single nanorod, demonstrating the diversity and flexibility of the multilayer structures we designed. By changing the doping locations (marked by a, b and c) as well as the combinations of Yb/Tm, Yb/Er and Yb/Ho pairs, in all, 64 (that is, 4×4×4) kinds of KSc_2F_7 nanorods with diverse UC emission patterns and lifetime outputs can be obtained by adopting the multilayer-structured design strategy we specially designed.

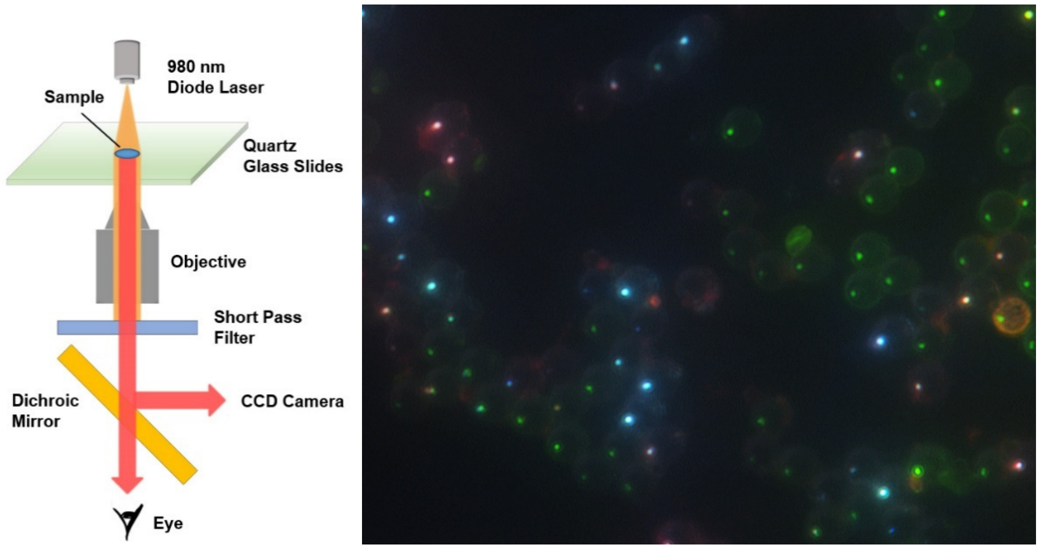

Fig. S19 Schematic illustration of the experimental setup (left) for UC luminescent imaging of the multilayer-structured KSc_2F_7 nanorods tagged polystyrene microbeads. A 980-nm diode laser was used as excitation source. The UC emission light was separated from the excitation light by a dichroic mirror (DMSP805R), and shot pass filter of 750 nm (FESH0750) was placed in the front of the detector to filter the excitation light. The UC luminescence image was recorded by a Nikon ECLIPSE Ti-U microscope equipped with Nikon digital sight DS-Ri1 CCD imaging system. UC luminescence photograph (right) of the nanorod-tagged polystyrene microbeads at low magnification showing that multicolor UC emissions can be simultaneously imaged under 980-nm diode laser excitation. Note that the blue, cyan, green, yellow and red colors were generated from Tm, Er and Ho ions doped in different KSc_2F_7 nanorods with different multilayer structures.

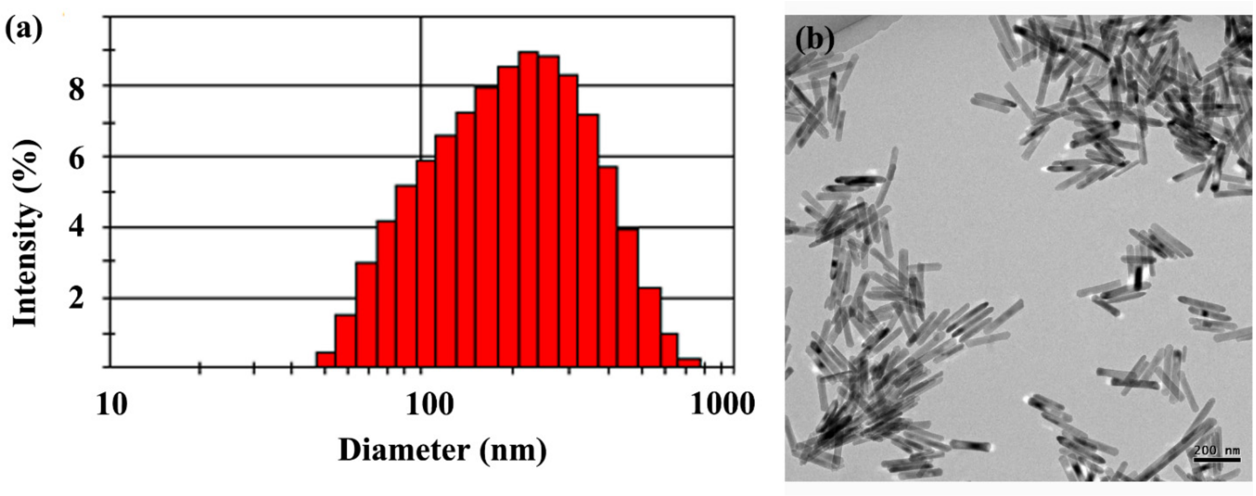

Fig. S20 (a) Size distribution for water-soluble Tm@Er@Ho@Pure nanorods obtained from DLS analysis, and their corresponding b) TEM image when dispersed in aqueous solution. The average hydrodynamic diameter for the water-soluble Tm@Er@Ho@Pure nanorods dispersed in aqueous solution was determined to be ~224 nm, which is basically consistent with that of the as-synthesized KSc_2F_7 nanorods with an average length of ~241 nm from the TEM images and thereby suggests the good dispersibility for the water-soluble KSc_2F_7 nanorods in aqueous solution.

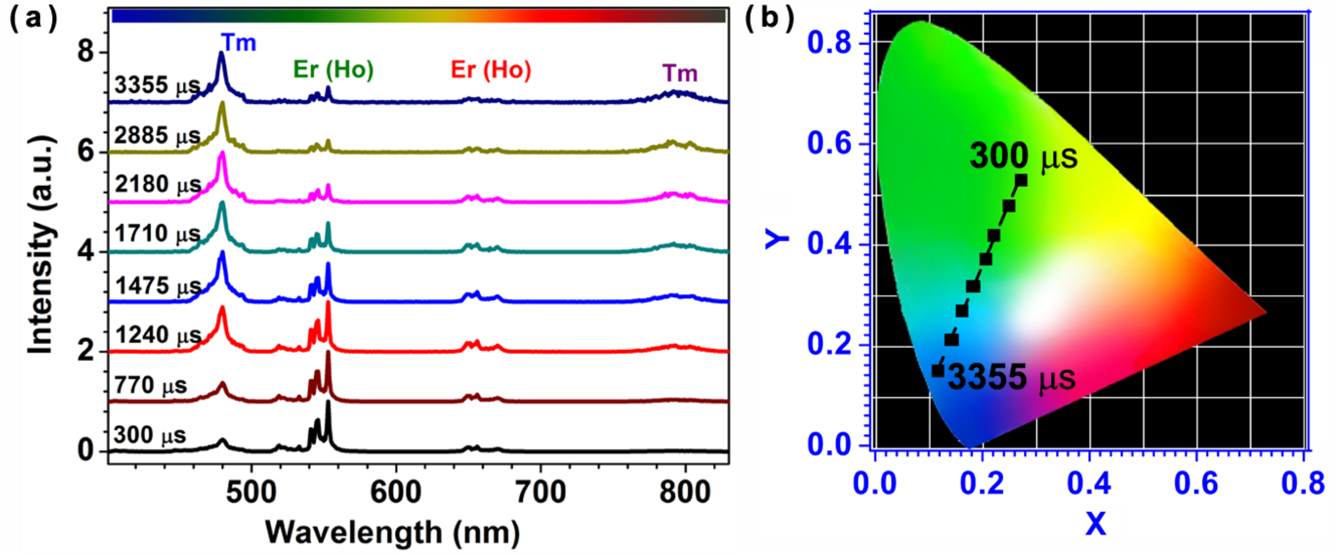

Fig. S21 (a) Time evolution of UC emission spectra for Tm@Er@Ho@Pure nanorods when excited at 980 nm and (b) their corresponding CIE chromaticity coordinates. Due to the different UC lifetimes of Tm, Er and Ho emissions in Tm@Er@Ho@Pure nanorods, individual UC emissions of Tm, Er and Ho ions can be easily distinguished from each other by setting an appropriate delay time in the time-resolved luminescence detection. As shown in Figure S19a, in short delay time (0-1240 μs), the whole UC emission spectra are dominated by the short-lived green emissions of Er and Ho ions (centered at 554 nm) upon excitation at 980 nm, while the long-lived blue emissions of Tm are prominent when the delay time is longer than 1475 μs, inducing a significant UC color change from green to cyan and to blue as revealed by their corresponding CIE chromaticity coordinates (Figure S19b). This feature makes these multilayer-structured KSc_2F_7 nanorods particularly suitable as time-resolved UC barcodes for advanced anti-counterfeiting in view of the tunable UC color outputs as a function of the detection time.

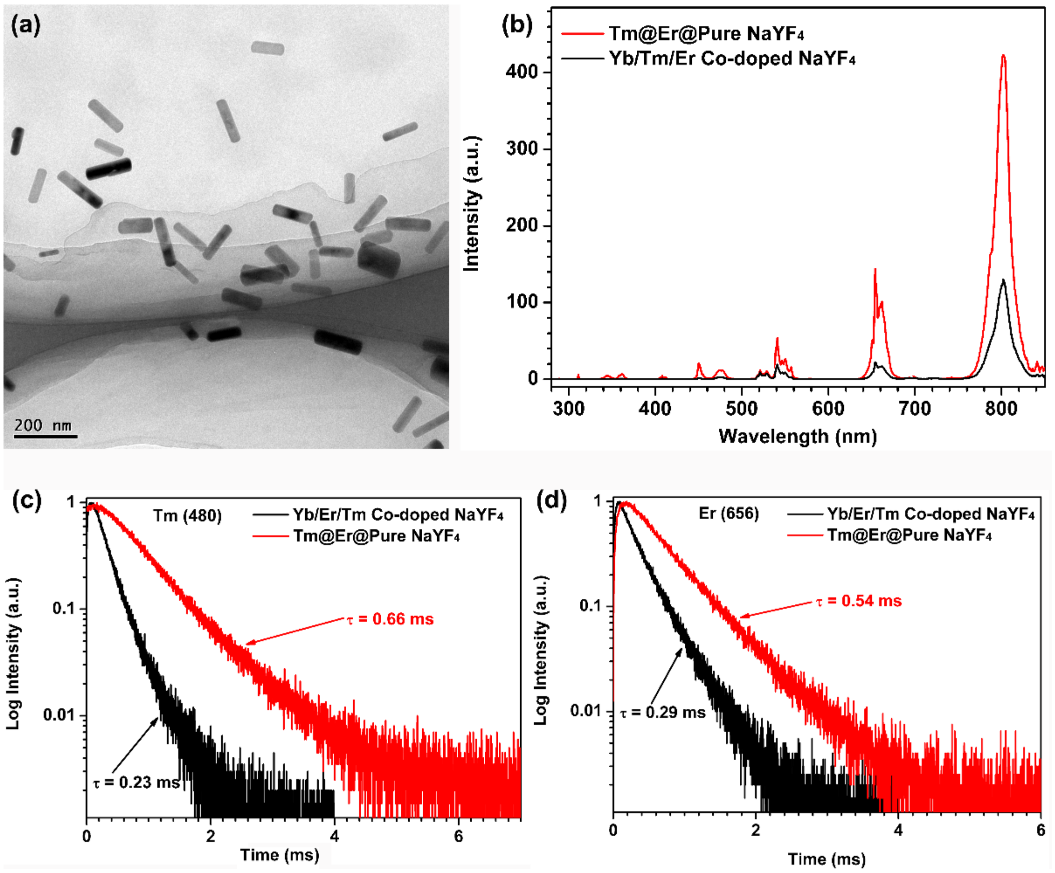

Fig. S22 (a) TEM image for multilayer-structured hexagonal phase $NaYF_4$:Yb/Tm/Gd@$NaYF_4$:Gd@$NaYF_4$:Yb/Er/Gd@$NaYF_4$:Gd (Tm@Er@pure $NaYF_4$) nanorods synthesized by a slightly modified literature procedure via hydrothermal reaction,[5] showing that the as-synthesized Tm@Er@pure $NaYF_4$ nanorods have a rod shape with an average length of 150 ± 30 nm and a diameter of 30 ± 10 nm. The doping concentrations of Yb, Er, Tm and Gd ions were determined to be 20, 2, 1 and 25 mol%, respectively, in $NaYF_4$ nanorods. (b) Comparison of UC luminescence spectra for Tm@Er@pure $NaYF_4$ and Yb/Tm/Er co-doped $NaYF_4$ nanorods. UC luminescence decays from (c) $^1G_4$ of Tm and (d) $^4F_{9/2}$ of Er by monitoring their UC emissions centered at 480 and 656 nm, respectively, upon excitation with a 980-nm pulsed laser. Similar to the cases of Tm@Er@Pure KSc_2F_7 nanorods we synthesized via a modified stepwise oriented epitaxial growth method, enhanced UC luminescence coupled with prolonged UC lifetimes were also detected in Tm@Er@pure $NaYF_4$ nanorods. These results clearly demonstrate that the design strategy we adopted for inhibiting CR-ETs of multiple Ln^{3+} ions can be readily generalized for the most promising UC luminescent system of β-$NaYF_4$, thus revealing the universality of the multilayer-structured strategy we specifically designed for highly enhanced UC luminescence.

Supplementary Table

Table S1. Absolute UC QYs in Ln-doped UC nanoparticles (UCNPs). All the values were acquired under NIR laser excitation at 980 nm unless for specific designation. Note that the high UC QYs were only achieved in the cases of Yb/X pair singly-doped NPs, and such a high UC QYs up to 3.9% had not yet been achieved in multiple Yb/X pairs co-doped UCNPs before.

Samples	Size (nm)	Power density (W cm^{-2})	QY (%)	Ref.
Tm@Er@Ho@Pure nanorods	~245 nm in length, ~25 nm in diameter	50	3.9 ± 0.2	this work
Tm@Er@Ho@Pure nanorods	~245 nm in length, ~25 nm in diameter	20	3.5 ± 0.2	
Tm@Er@Ho nanorods	~190 nm in length, ~22 nm in diameter	50	1.9 ± 0.1	
Yb/Tm/Er/Ho co-doped KSc_2F_7 nanorods	~245 nm in length, ~25 nm in diameter	50	0.2 ± 0.1	
$NaYF_4$:Yb/Er bulk	>> 100 nm	20	3.0 ± 0.3	Ref.[6]
$NaYF_4$:Yb/Er@$NaYF_4$ core-shell NPs	~30 nm	150	0.3 ± 0.1	
$NaYF_4$:Yb/Er NPs	~100 nm	150	0.3 ± 0.1	
$LiYF_4$:Er NPs	85	150 (λ_{ex}= 1500 nm)	1.2	Ref.[7]
$NaYF_4$:Yb,Tm@$NaYF_4$ core-shell NPs	42	78	3.5	Ref.[8]
$NaYF_4$:Yb,Tm NPs	33	3.8	0.45	Ref.[9]

$LiLuF_4$:Yb,Er (or Tm) NPs	28	127	0.11 (0.61)	Ref.[2]
$LiLuF_4$:Yb,Er@$LiLuF_4$ core-shell NPs	50	127	5.0	
$LiLuF_4$:Yb,Tm@$LiLuF_4$ core-shell NPs	50	127	7.6	
$NaGdF_4$:Yb/Er@$NaYF_4$ core-shell NPs	~13	50	0.89 ± 0.05	Ref.[10]

References

1 J. E. Roberts, *J. Am. Chem. Soc.* 1961, **83**, 1087.
2 Y. Liu, S. Zhou, Z. Zhuo, R. Li, Z. Chen, M. Hong, X. Chen, *Chem. Sci.* 2016, **7**, 5013.
3 Y. S. Liu, D. T. Tu, H. M. Zhu, X. Y. Chen, *Chem. Soc. Rev.* 2013, **42**, 6924.
4 Y. Lu, J. Zhao, R. Zhang, Y. Liu, D. Liu, E. M. Goldys, X. Yang, P. Xi, A. Sunna, J. Lu, Y. Shi, R. C. Leif, Y. Huo, J. Shen, J. A. Piper, J. P. Robinson, D. Jin, *Nat. Photon.* 2014, **8**, 32.
5 Y. H. Zhang, L. X. Zhang, R. R. Deng, J. Tian, Y. Zong, D. Y. Jin, X. G. Liu, *J. Am. Chem. Soc.* 2014, **136**, 4893.
6 J. C. Boyer, F. C. J. M. van Veggel, *Nanoscale* 2010, **2**, 1417.
7 G. Y. Chen, T. Y. Ohulchanskyy, A. Kachynski, H. Agren, P. N. Prasad, *Acs Nano* 2011, **5**, 4981.
8 C. T. Xu, P. Svenmarker, H. C. Liu, X. Wu, M. E. Messing, L. R. Wallenberg, S. Andersson-Engels, *Acs Nano* 2012, **6**, 4788.
9 H. C. Liu, C. T. Xu, D. Lindgren, H. Y. Xie, D. Thomas, C. Gundlach, S. Andersson-Engels, *Nanoscale* 2013, **5**, 4770.
10 X. M. Li, R. Wang, F. Zhang, D. Y. Zhao, *Nano Lett.* 2014, **14**, 3634.
